# Lung cancer associated with combustion particles and fine particulate matter (PM_2.5_) - The roles of polycyclic aromatic hydrocarbons (PAHs) and the aryl hydrocarbon receptor (AhR)

**DOI:** 10.1016/j.bcp.2023.115801

**Published:** 2023-09-09

**Authors:** Jørn A. Holme, Jan Vondráček, Miroslav Machala, Dominique Lagadic-Gossmann, Christoph F.A. Vogel, Eric Le Ferrec, Lydie Sparfel, Johan Øvrevik

**Affiliations:** a Department of Air Quality and Noise, Division of Climate and Environmental Health, Norwegian Institute of Public Health, PO Box PO Box 222 Skøyen, 0213 Oslo, Norway; b Department of Cytokinetics, Institute of Biophysics of the Czech Academy of Sciences, 61265 Brno, Czech Republic; c Department of Pharmacology and Toxicology, Veterinary Research Institute, 62100 Brno, Czech Republic; d Univ Rennes, Inserm, EHESP, Irset (Institut de Recherche en Santé, Environnement et Travail), UMR_S 1085, F-35000, Rennes, France; e Department of Environmental Toxicology and Center for Health and the Environment, University of California, Davis, CA 95616, USA; f Department of Biosciences, Faculty of Mathematics and Natural Sciences, University of Oslo, PO Box 1066 Blindern, 0316 Oslo, Norway; g Division of Climate and Environmental Health, Norwegian Institute of Public Health, PO Box 222 Skøyen, 0213 Oslo, Norway

**Keywords:** Air pollution, Diesel exhaust, Smoking, Occupational exposure, Carcinogenesis, Genotoxicity, Inflammation, Tumor promotion, Tumor microenvironment, Tumor metastasis

## Abstract

Air pollution is the leading cause of lung cancer after tobacco smoking, contributing to 20% of all lung cancer deaths. Increased risk associated with living near trafficked roads, occupational exposure to diesel exhaust, indoor coal combustion and cigarette smoking, suggest that combustion components in ambient fine particulate matter (PM_2.5_), such as polycyclic aromatic hydrocarbons (PAHs), may be central drivers of lung cancer. Activation of the aryl hydrocarbon receptor (AhR) induces expression of xenobiotic-metabolizing enzymes (XMEs) and increase PAH metabolism, formation of reactive metabolites, oxidative stress, DNA damage and mutagenesis. Lung cancer tissues from smokers and workers exposed to high combustion PM levels contain mutagenic signatures derived from PAHs. However, recent findings suggest that ambient air PM_2.5_ exposure primarily induces lung cancer development through tumor promotion of cells harboring naturally acquired oncogenic mutations, thus lacking typical PAH-induced mutations. On this background, we discuss the role of AhR and PAHs in lung cancer development caused by air pollution focusing on the tumor promoting properties including metabolism, immune system, cell proliferation and survival, tumor microenvironment, cell-to-cell communication, tumor growth and metastasis. We suggest that the dichotomy in lung cancer patterns observed between smoking and outdoor air PM_2.5_ represent the two ends of a dose–response continuum of combustion PM exposure, where tumor promotion in the peripheral lung appears to be the driving factor at the relatively low-dose exposures from ambient air PM_2.5_, whereas genotoxicity in the central airways becomes increasingly more important at the higher combustion PM levels encountered through smoking and occupational exposure.

## 1. Introduction

Lung cancer has long been recognized as one of the leading causes of cancer‑associated mortality [1–3]. It is a complex process which develops slowly over time, and consequently, most people diagnosed with lung cancer are 65 or older [4]. Central steps in the development include tumor initiation, tumor formation and progression, matrix remodeling, intravasation, extravasation and metastasis [5]. Each step is determined by genetic predispositions and mutations acquired over an individual’s lifetime due to endogenous processes, lifestyle factors and/or environmental exposures.

Although smoking remains the biggest risk factor for lung cancer, about 25% of the cases are not attributable to tobacco [6]. The Global Burden of Disease (GBD) Project has estimated that 19% of lung cancer deaths are associated with exposure to air pollution making it the second largest risk factor [7]. The majority of this is mainly attributed to fine particulate matter, PM_2.5_ (with particle aerodynamic diameter of less than 2.5 μm), derived from combustion sources such as traffic exhaust, coal and biomass burning, and industrial activities [7]. Outdoor air PM and diesel exhaust particles (DEP) have been classified as Group 1 known human carcinogens by the International Agency for Research on Cancer [8,9]. Other combustion PM sources such as cigarette smoke [10,11] and indoor combustion of coal [12] have also been classified as Group 1 human carcinogens, while emissions from the burning of biomass/wood have been classified as a Group 2A (probable) human carcinogen [12]. Epidemiological studies indicate that PM_2.5_ exposure may increase both the incidence and mortality rates associated with lung cancer [13], and also decrease the survival time of patients with lung cancer [14]. Several studies have also reported an increased association between living near busy roadways and lung cancer incidence and mortality in Asia, Europe and North-America, pointing towards a central role of direct exposure to combustion emissions from road vehicles such as ultrafine particles and/or volatile/semi-volatile organic compounds [15–19].

The causal links between combustion PM exposure and lung cancer development are further supported by both *in vitro* and *in vivo* studies [8,9,20,21]. Combined epidemiological and experimental studies have provided essential information on cancer acquisition hallmarks including genetic instability, sustained proliferative signaling, insensitivity to antigrowth signals, resistance to cell death, replicative immortality, replicative immortality, dysregulated metabolism, tumor promoting inflammation, angiogenesis, tissue invasion and metastasis [5,22]. Thus, modifications of a variety of biological processes seem to contribute to the carcinogenic effects of PM_2.5_.

Combustion-derived PM typically consists of aggregates of smaller carbon particles with mixtures of organic chemicals adhered to their surface [23]. Their carcinogenic properties have largely been attributed to extractable organic material (EOM) and the content of polycyclic aromatic hydrocarbons (PAHs) [24]. PAHs are a highly diverse group of chemicals originating from combustion of organic materials. Numerous PAHs are considered important air pollutants and particle toxicants. Some of them are classified either as carcinogenic or probably carcinogenic to human respiratory organ [8,9,25–27]. Other effects that have been linked to PAHs exposure via PM_2.5_ inhalation are impairment of respiratory functions, exacerbation of asthma and increased morbidity/mortality of obstructive lung diseases [28].

Several PAHs are considered complete carcinogens contributing to both tumor initiation and promotion [5,25,29]. Nevertheless, the carcinogenicity of PAHs is most often linked to their metabolism and genotoxicity: the formation of reactive electrophilic metabolites forming covalent DNA adducts leading to mutations in oncogenes and tumor suppressor genes [30]. Importantly, the mutagenic signatures of EOM of cigarette smoke, combustion PM and air pollution PM resemble the mutation pattern of benzo[*a*]pyrene (B[a]P), and the same mutations are also found in lung cancers from smokers and people exposed to high levels of combustion aerosols from indoor use of smoky coal or in occupational settings [24].

The metabolism and genotoxicity of PAHs are largely regulated by the aryl hydrocarbon receptor (AhR) through transcriptional control of xenobiotic metabolizing enzymes [31]. The AhR, which is the main cellular sensor of PAHs and other aromatic compounds, is a basic helix-loop-helix PAS transcription factor, expressed in almost all tissues including a number of lung cell types such as bronchial epithelial cells, alveolar type II cells, club (Clara) cells, endothelial cells and macrophages [32,33]. The prototypic genes regulated by AhR are the cytochrome P450 (CYP) family 1 members CYP1A1 and CYP1B1. While the CYP enzymes are generally considered to be important detoxification enzymes, CYP1A1 and CYP1B1are also involved in the metabolic activation of chemicals such as B[*a*]P into its ultimate carcinogen B[*a*]P-7,8-dihydrodiol-9,10-epoxide (BPDE) [34]. In line with this, the AhR appears to be essential for the carcinogenic effects of B[*a*]P [35,36].

While PAH-induced genotoxicity may be central to lung cancer development in smokers, it has become increasingly clear that the pattern of mutations and lung cancer subtypes in never-smokers are distinctly different [6,24,37]. Lung cancer in never-smokers rather appears to derive from naturally occurring mutations [37]. As PM_2.5_ is regarded as the main cause of lung cancer in never-smokers, it is possible that the carcinogenic effects of air pollution differ from those of smoking and that PAH-induced genotoxicity is of lesser importance. In support of this, a recent study suggests that tumor promotion is the main driver of air pollution-induced lung cancers [38]. However, this does not exclude other roles of PAHs and AhR in cancer development, which extends far beyond metabolic activation and genotoxic effects of PAHs. One of the best described roles of AhR is the tumor promoting action of 2,3,7,8-tetrachlorodibenzo-*p*-dioxin (TCDD) [39–42]. In fact, AhR appears to be involved in all of the major stages in cancer development, including cancer initiation, promotion, progression, invasion, and metastasis. It has thus emerged as a regulator of malignant cell progression and immune evasion associated with poor cancer outcomes [43,44].

**Fig. 1.**
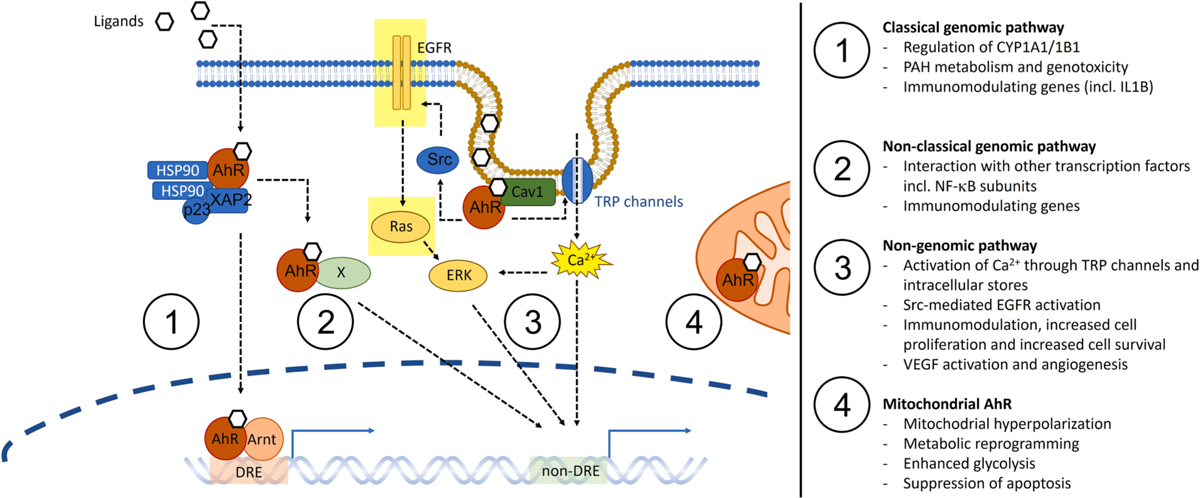
Overview of the main signaling pathways of AhR and the cancer-related responses regulated by these. AhR may induce effects through at least four different signaling modes. In the classical genomic pathway (1), inactive AhR resides in the cytosol bound to heat-shock protein 90 (HSP90), XAP2, and p23 proteins. Upon ligand activation, AhR translocates to the nucleus, dimerizes with its binding partner Arnt, and the AhR:Arnt dimer binds to dioxin response elements (DREs) in the regulatory region of target genes. The prototypical genes activated are the CYP1A1/−1B1 enzymes, which may metabolize PAHs into genotoxic metabolites. However, a number of genes express DRE sites and are affected by classical AhR signaling, including IL1B and other proinflammatory cytokines. AhR may also dimerize with other binding partners (X) such as NF-κB subunits through non-classical genomic signaling (2), activating alternative binding sites and regulate other genes including various immunomodulating factors. A subfraction of AhR appears to be localized in close connection interacting with caveolin-1 (Cav1) in caveolae, acting as a cytosolic signaling molecule in the so-called non-genomic pathway (3). Non-genomic AhR signaling regulates rapid activation of Ca2 + signaling from transient receptor potential (TRP) channels and intracellular stores, and Src-mediated activation of EGFR-RAS-ERK signaling which may regulate cell proliferation, cell survival, angiogenesis and immunomodulating responses. Importantly mutations in the KRAS (Ras) and EGFR genes are characteristic of lung cancers in smokers and never-smokers, respectively, underscoring the potential importance of the non-genomic pathway. Another subfraction of AhR has been localized in the inter-membrane space of mitochondria, mitochondrial AhR (4), and may regulate mitochondrial polarization, metabolic reprogramming, glycolysis and apoptosis, which is also associated with lung cancer development.

In light of the emerging evidence suggesting that lung cancer development from air pollution differs from what is seen in smokers [6,37,38], this review aims to address the many-faceted roles of PAHs and AhR in cancer development associated with combustion particle exposure (Fig. 1). We will discuss their potential involvement in all stages of carcinogenesis, from DNA damage to promotion, progression, invasion, and metastasis, and whether some of the differences observed between smoking and urban air PM_2.5_ may rather be a matter of the dose.

## 2. PM_2.5_, sources, and PAH characteristics

Potential mediators/modulators of the carcinogenic effects of PM_2.5_ and combustion-derived PM include the particle shape and size, surface reactivity (charge and presence of reactive groups including redox-active transition metals) and adherence of various organic components (PAHs, PAH-quinones and bacterial endotoxins) [45]. While the levels of organic chemicals are often found to be in the range 20–30% of total particle mass, it may reach as much as 90% [46]. The specific composition and the relative amount of chemicals attached to PM_2.5_ are highly dependent on sources, including combustion technology and fuel burned. Traditionally, diesel engine particles (DEP) have received most attention, and DEP emissions can be distinguished from gasoline emissions and wood smoke particles (WSP) by a high level of unresolved alkanes [47,48]. DEP also contained higher levels of alkylated and nitrated PAHs (alkyl-PAHs and nitro-PAHs) compared to other combustion PM [25,47]. By contrast, WSP may contain somewhat higher levels of oxygenated and hydroxylated PAHs (oxy-PAHs and hydroxy-PAHs), as compared to traffic emissions [47,49].

The relative contribution of different sources to PAHs measured on PM_2.5_ is changing as combustion technologies develop. The introduction of ever improved emission aftertreatment, such as EURO-classified diesel particulate filter (DPF) has considerably reduced both PM and PAH emissions from modern diesel vehicles, and today light-duty gasoline vehicles represent the dominating PAH source from traffic [50]. Notably, exhaust from modern gasoline vehicles contains very low levels of PM, and the majority of organic chemicals emitted occur in the gas phase, and then condenses to form secondary aerosols in the atmosphere [51,52]. Nevertheless, traffic emissions remain a major source of increased urban air PAH levels. Recent studies of road tunnels PM suggested that PAHs on traffic PM_2.5_ were primarily attached to aggregates of ultrafine PM originating from the combustion of transportation fuel [53,54]. More US EPA PAHs have been found in the ultrafine and fine PM (PM_2.5_) samples than in the coarse PM, which to a large degree seem to originate from non-combustion sources such as bitumen and tires [54]. Phenanthrene > pyrene > fluoranthrene were the most abundant species. However, high amounts of PAHs with 4 rings (benz[*a*]anthracene, chrysene) and 5 rings (B[*a*]P, benzo[*e*]pyrene, benzo[*k*]fluoranthene, benzo[*j*]fluoranthene, dibenz[*a,h*]anthracene), as well as the strong mutagen cyclopenta[*c,d*]pyrene were also found in these combustion PM samples [54]. In addition to PAHs, oxygenated (oxy-PAHs; 9H-fluoren-9-one and anthracene-9,10-dione) and nitrated (nitro-PAHs; 1-nitronaphtalene, 9-nitroanthracene and 1-nitropyrene) PAH derivatives from diesel engine emissions are found both in ultrafine and fine PM [9,54,55].

In general, specific profiles of PAHs associated with PM of various origin can lead to distinct toxic and carcinogenic potencies being linked with PM exposure. These may include both genotoxic and non-genotoxic modes of action, as discussed further in sections to follow. Airborne PM usually contain relatively high levels of carcinogenic priority PAHs (chrysene, benzo[*b*]fluoranthene, benzo[*k*[fluoranthene, B[*a*]P and indeno[*1,2,3-cd*]pyrene). Mixtures of PAHs associated with DEP have significantly higher total sum of PAHs in comparison to airborne PM samples; specifically, they contain higher levels of fluoranthene, pyrene, chrysene, benzo[*j*]fluoranthene, benzochrysenes and monomethylated anthracenes, phenanthrenes, pyrenes and benz[*a*]anthracenes [56]. DEP also contains high concentrations of nitro-PAHs formed through electrophilic substitution in the presence of NO_2_ [57]. Some nitro-PAHs such as 1-nitropyrene (1-NP) are formed mainly during the combustion process and have been suggested as a marker of DEP exposure, while others are formed through atmospheric processes between NO_2_ and gas-phase PAHs [57,58].

The PAHs composition in urban air PM_2.5_ does not depend only on the combustion sources, but it is largely affected by the environmental conditions. Volatility is reduced by size; therefore, smaller PAHs (four or fewer aromatic rings) are to a greater extent found in the gas phase, while high-molecular weight PAHs (five or more aromatic rings) are mainly detected on the particle [25]. However, as low-molecular weight PAHs are usually formed to a much greater extent than the larger PAHs, they also tend to be the dominating PAHs bound to PM. Accordingly, levels of e.g., phenanthrene and pyrene on DEP and urban air PM_2.5_ exceed the level of B[*a*]P [25,59]. The amount and type of PAHs being present on PM_2.5_ are further modified by ambient air temperature and photooxidation processes. As condensation and evaporation processes are directly regulated by temperature, higher levels of PAHs condense onto ambient particulates at low temperatures. The total PAH content in urban air PM_2.5_ can therefore be an order of magnitude higher in winter as compared to summer, and the relative amount of different PAH species may also change due to seasonal variation in sources, such as residential heating and forest fires [60–63]. Furthermore, photooxidation leads to formation of oxy-PAHs which contributes to SOA formation by reducing the vapor pressure compared to their parent PAHs and increasing the condensation process [64,65]. Importantly, while photooxidation of PAHs may increase their redox and direct mutagenic activities, it also leads to a reduced affinity towards AhR [66–68]. However, photo-oxidation also increases water solubility, which has been suggested to limit the bioavailability of oxy-PAHs [69].

## 3. Lung cancer

There are two main histopathological lung cancer groups: non-small cell lung cancer (NSCLC) [70] and small-cell lung cancer (SCLC) [71]. NSCLC accounts for 80% of the lung cancer in humans [72]. The majority of NSCLC are adenocarcinomas (ADC), the other histopathological NSCLC subtypes are squamous cell carcinoma (SCC) and large cell carcinoma. Although the cellular origin(s) of lung cancer remain largely unknown it has been speculated that different histopathological subtypes arise from distinct cells localized in defined microenvironments [73]. Due to their proximal-to-distal distribution pattern, SCC is often thought to arise from the proximal airway and ADC from more distal locations [74].

Lung cancers develop through a process involving multiple genetic and epigenetic alterations in the cells of origin(s). Examples of genes that have been linked to lung carcinogenesis are oncogenes/growth promoting proteins (e.g., v-Ki-ras2 Kirsten rat sarcoma viral oncogene homolog [KRAS], epidermal growth factor receptor [EGFR], tyrosine protein kinase c-Src, B-Raf proto-oncogene [BRAF], mitogen activated protein/extracellular regulated kinase [MEK-1], human epidermal growth factor receptor 2 [HER2], hepatocyte growth factor receptor [MET], anaplastic lymphoma kinase [ALK], and rearranged during transfection [RET]). Lung carcinogenesis also typically involves inactivation of tumor suppressor genes/proteins (e.g., TP53/p53, phosphatase with tensin homology [PTEN], and liver kinase B1 [LKB-1]) [30,75]. Mutations in the TP53 gene are frequent in almost all types of cancers [76], and they are present in approximately 50% of all NSCLC cases [77]. A frequent transversion, G:C to T:A, is correlated with exposure to carcinogens found in tobacco [78]. At several TP53 mutational hotspots, such as codons 248 and 273, a large fraction of the mutations is G to T events in overall lung cancers, while almost exclusively G to A transitions are found in non-tobacco-related cancers [6]. There seems to be a strong coincidence of G to T transversion hotspots in lung cancers and sites of preferential formation of PAH adducts along the TP53 gene [24,78].

EGFR and KRAS are two other frequently mutated genes in lung cancer. The EGFR receptor regulates cell survival and proliferation, and it is overexpressed in 50% of lung cancers. KRAS belongs to the Ras family of small GTPases which regulates downstream signaling of EGFR to the extracellular regulated kinases (ERK1/2), which is central for the cell growth and proliferation [6]. The EGFR-Ras-ERK1/2 pathway also regulates several proinflammatory genes which may affect the tumor microenvironment as discussed later. KRAS mutations are frequent in smokers but occur in only 5 to 10% of lung cancers in never- or light-smokers [79–81]. The KRAS mutations are often generated by G to T transversions associated with tobacco use and PAH exposure, and they lead to loss of the GTPase activity which is necessary for the inactivation of Ras in the GDP-bound form leaving the protein constitutively active [6]. EGFR mutations, on the other hand, are present in 15 to 50% of NSCLC patients from never-smokers, and the mutational pattern seems to be dominated by transition mutations (G to A) [80–82]. Deletion in exon 19 and the single amin acid substitution L858R in exon 21 (replacing leucin with arginine in codon 858) of the EGFR gene account for about 85% of observed EGFR mutations in NSCLC. This destabilizes the inactive form of the receptor leading to increased dimerization and activation compared to wildtype EGFR [83]. As EGFR and Ras are part of the same signaling pathway, both mutations target the peripheral airways and give rise to ADC [6]. However, while lung cancer in never smokers with EGFR driver mutations may be sensitive to EGFR tyrosine kinase inhibitor (EGFR TKI) treatment, lung cancers in smokers with KRAS mutations are often resistant to EGFR TKI treatment underscoring that upstream activation of EGFR is not necessary for the Ras activity in these patients [6]. Furthermore, while smoking tends to induce SCLC and SCC in the central airways, ADC in the peripheral regions is the most prevalent lung cancer type in never-smokers [6]. Thus, both the mutation pattern and lung cancer subtypes seem to be distinctly different in smokers and never-smokers.

Tissue stem cells are attractive candidates for cellular origin of cancer, as their long lifespan allows them to accumulate genetic mutations essential for cancer development [84]. A subtype of lung adenocarcinoma with KRAS mutations has been suggested to evolve from airway epithelium, having a distinct differentiation pattern with suppression of ciliated and exocrine bronchiolar cell (Clara cell)-related genes [85]. Based on histological observations and studies with genetically engineered mouse models, alveolar type 2 (AT2) cells have been hypothesized to be the cells of origin of another subpopulation of lung adenocarcinoma [86].

More recently, high-resolution mutational profiles of lung epithelial cells exposed to individual tobacco smoke chemicals support a role for PAHs like B[*a*]P [87]. Such studies have revealed that lung cancer with metastasis is a process not only linked to lung cancer stem cells transformation and epithelial-mesenchymal transition (EMT), but also to modifications of the tumor microenvironment of lung cancer [88,89] and mechanisms linked to angiogenesis and lymph angiogenesis [90]. Central influencing factors of lung cancer also include many noncoding RNAs (ncRNAs, miRNA) [91].

## 4. Lung cancer induced by combustion PM/PAHs

PM_2.5_ exposure from polluted air is the main risk factor for lung cancer in never-smokers, which predominately develops as ADC with EGFR driver-mutations in the peripheral lung [6]. A genomic analysis found that most of these tumors appeared to originate from natural mutations accumulating with age [37]. This implies that mutagens and genotoxic effects may not be the main drivers of air pollution induced lung cancer. While the frequency of EGFR-driven lung cancers seems to increase with increasing PM_2.5_ exposure, there are no changes in the accompanying EGFR mutation pattern, indicating that PM_2.5_ primarily induces ADC through promotion [38]. Studies in mouse models and *in vitro* support and extend this hypothesis by suggesting that macrophages exposed to PM_2.5_ induced a progenitor-like state in AT2 cells containing natural acquired mutated EGFR (L858R). Furthermore, interleukin (IL)-1β seems to be required for the promotion phase [38]. This aligns with earlier findings by Riva et al (2020) reporting that only 3 out of 20 tested suspected human carcinogens induced carcinogen-specific mutations in mice [92]. These authors therefore hypothesized that “*key driver mutations are likely to be acquired through endogenous mutagenic processes rather than by the direct action of chemical exposures on the genome*” and further speculated that inflammation could be a driving factor for tumor promotion [92].

Notably, IL-1β release and inflammation are also considered the driving force in silica- and asbestos-induced lung cancer [93,94], but EGFR mutations appear to be less frequent in never-smokers occupationally exposed to such mineral particles [95]. By contrast, never-smokers occupationally exposed to diesel exhaust particles and PAHs had equal or higher frequency of EGFR mutations compared to controls [95]. This indicates that additional mechanisms and properties associated with combustion particle exposure such as PAHs, may be necessary to promote EGFR-driven lung cancers. In line with this, an important role of IL-1β has been identified in inflammation-induced and AhR-dependent tumor promotion of lymphoma in mice [96].

Based on the differences in lung cancer subtypes and mutation spectra found in smokers versus never-smokers, it has been proposed that lung cancer in never-smokers is “a different disease” than lung cancer in smokers [6]. However, 8% of lung cancers in smokers lack evidence of smoking-induced mutagenesis, suggesting that also smoking may promote cancer through non-genotoxic mechanisms [97]. The marked reduction in risk of lung cancer following smoke cessation further points to a major role for tumor promotion also in smoking-induced cancers [24]. Moreover, lung cancer development from secondhand smoke (SHS) resembles never smokers in that ADC also seem to be the predominant cancer subtype and tobacco-induced mutations are lacking [37,98].The differences observed between smoking versus air pollution and SHS may rather be a matter of exposure dose. In further support of this, a *meta*-analysis of 16,000 lung cancer cases concluded that occupational exposure to diesel exhaust were associated with all lung cancer types, but the dose-dependency were much stronger for SCC than for ADC [99]. In other words, the ratio of SCC:ADC increased at higher DEP exposure levels. In line with this, the SCC:ADC ratio has been reported to be almost 3:1 in smokers who are exposed to very high PM doses, but inversed (more than 1:3) in never-smokers only exposed to low PM concentrations through ambient air [6]. Indoor exposure to smoky coal, which is considered to be 100-fold more carcinogenic than cigarette smoke and represents a high-dose exposure to combustion particles compared to outdoor air PM_2.5_ levels, has also been reported to cause an overrepresentation of G to T transversions in the TP53 gene similar to what is found in smokers and PAH exposed workers [6,12,24]. A systematic review of indoor exposure to coal and biomass smoke also concluded that the odds ratio (OR) of developing SCC was higher than the OR for developing ADC (3.58 vs. 2.33), again pointing towards a pattern of lung cancer subtypes more in the direction of smoking [100].

Occupational exposure to diesel exhaust and indoor exposure to solid fuel smoke represents much higher combustion PM exposures levels than those that are normally encountered in outdoor environments. Thus, low dose exposure to combustion particles appears mainly to induce ADC in the peripheral lung regions, but as concentrations increase, the risk of SCC development in the central airways increases much more than ADC, and becomes the dominant cancer type [99]. This apparent dose-dependent shift in lung cancer subtypes associated with various combustion PM exposures could likely be related to a dose-dependent increase in cilia dysfunction and impairment of particle clearance, as observed with tobacco smoking [101,102]. Thus, increased inhalation of combustion PM may exponentially increase the effective PM dose on bronchial epithelial cells by impairing the mucociliary clearance of deposited particles. This could explain the increased risk of SCC development from smoking, occupational diesel exposure and indoor air solid fuel smoke, compared to ADC [99].

At higher exposure doses, combustion PM-induced genotoxicity also appears to become more important. A number of experimental studies in rodents have proven the carcinogenic potency of PM and/or extractable organic matter (EOM) from a variety of combustion and urban air PM (primarily PM_2.5_) [8,9,12,103,104]. In a recent review, the carcinogenic potency of EOM on Sencar mouse skin from a variety of combustion emissions, coal tar, and B[*a*]P were presented [24]. B[*a*]P was found to have the highest carcinogenic potential. Most interestingly, the carcinogenic potency of EOM of urban air pollution as well as diesel and gasoline exhaust could be at least two orders of magnitude higher than EOM for tobacco smoke. EOM of ambient air PM, various combustion particles and cigarette smoke predominately induced G to T transversion in the *Salmonella* (Ames) mutagenicity assay [24]. The mutation spectra observed in experimental studies therefore provide further support for the suggestion that air pollution and tobacco smoking could lead to comparable patterns of lung cancer development given exposure to comparable dose levels.

Based on the above, we hypothesize that the discrepancies in mutation patterns and cancer subtypes induced by smoking and air pollution (never smokers) reflect the two ends of a combustion PM dose–response continuum. We further suggest that the tumor promoting effects of combustion PM are most important for lung cancer development in the lower dose-range, but that their mutagenic effects become increasingly more important as the exposure dose increases. Accordingly, a series of *in vitro* studies performed in rat liver epithelial cells showed that only a few environmental PAHs and methylated PAHs elicit major genotoxic effects, determined as formation of stable DNA adduct production and/or p53 activation [105–109]. Dibenzo[*a,l*]pyrene (dibenzo[*def,p*]chrysene) has been observed to be the most potent genotoxin, while several PAHs, including benzo[*g*]chrysene, B[*a*]P, 5-methylchrysene, 1- and 3-methylbenzo[*a*]pyrene exhibited significant genotoxic potencies. Other PAHs and methyl-PAHs, including benz[*a*] anthracene, chrysene, benzo[*b*]- and benzo[*k*]fluoranthene and dibenzo [*a,h*]anthracene, induced only a moderate DNA adduct production in rat liver epithelial cells, and numerous other PAHs or monomethylated PAHs showed only a minimal or no genotoxicity potencies.

In line with this, the AhR-dependent proliferation of rat liver epithelial cells (WB-F344) exposed to EOM of urban dust PM (SRM1649a) has been reported to occur at an order of magnitude lower doses than DNA damage [110]. It was therefore suggested that non-genotoxic effects of AhR activation could be an important determinant of the effects of complex PAH mixtures from PM [110]. Transcriptional activation of AhR appears to be among the most sensitive, if not the most sensitive, endpoint induced *in vitro* by combustion PM in airway epithelial cells [111,112]. As discussed in this review, the role of AhR in lung cancer development extends far beyond the regulation of PAH metabolism, adduct formation, and genotoxicity. AhR plays a central role in cancer promotion pointing towards the non-genotoxic properties of PAHs. For instance, the AhR may directly regulate inflammatory responses and immune cells in the tumor microenvironment [113]. Moreover, nuclear AhR translocation, a hallmark of AhR activation, appears to be more common in female non-smokers with ADC, and it is associated with EGFR mutations [114–116]. At the same time, it seems that AhR may suppress KRAS-driven ADC [117]. These observations are in coherence with the suggested role of inflammation and tumor promotion in air pollution-induced lung cancer, as well as the long-recognized role of genotoxicity and mutagenesis in tobacco smoke-induced lung cancer.

It is pertinent to emphasize that the differences discussed here represent the main trends and patterns seen in lung cancer development. Some never-smokers also develop SCC and express KRAS-mutations and G to T transversion, while some smokers develop ADC and express EGFR mutations and G to A transversion [6,97]. Indeed, exposure to ambient air pollution and traffic emissions appear to be consistently associated with elevated urinary excreted PAH metabolites and biomarkers of genotoxicity, and also smoking may promote cancer development by increasing selection of cells with naturally acquired mutations [97,118,119]. In the following sections, we will discuss the potential involvement of AhR and PAHs at different stages of cancer development and progression.

## 5. Canonical AhR signaling and PAH metabolism

In the absence of a ligand, AhR resides in the cytosol as part of a multiprotein complex consisting of AhR-interacting protein (ARA9 or XAP2), a heat shock protein 90 dimer (Hsp90) and co-chaperone p23. In its major signaling route, the so-called canonical or classical AhR pathway, ligand-activated AhR dissociates from the multiprotein complex and translocates to the nucleus, where it dimerizes with the AhR nuclear translocator (Arnt). The AhR/Arnt heterodimer then binds to the so-called xenobiotic response elements (XREs), also known as dioxin response elements (DREs), in regulatory regions of target phase I and phase II genes (Fig. 1).

Several studies have revealed that PM_2.5_, more specifically the organic fractions of PM_2.5_/DEP may, through cell specific mechanism, form reactive metabolites and display CYP1A1 activation [21,22,120,121]. The AhR-dependent induction of CYP1A1 expression seems to represent a particular sensitive biomarker of DEP-exposure [111]. PAHs are among the most likely candidates contributing to such effects on combustion PM. Due to their lipophilic nature, PAHs may detach from the particle and diffuse across the plasma membrane into the cell. Highly depending on cell type, the PAHs may be metabolized to reactive electrophilic metabolites and/or give rise to a canonical AhR-response modifying PAH-metabolism. In the following we briefly summarize the main metabolic steps of PAHs using B[a]P as an example.

There are three major pathways for PAH/B[a]P metabolism, which are characterized by specific sets of enzymes: i) the cytochrome P450 (CYP)1A1/CYP1B1 and epoxide hydrolase, ii) aldo–keto reductase and iii) the CYP peroxidase enzyme [31,122]. i) In the CYP1A1/CYP1B1 and epoxide hydrolase pathway, PAHs/ B[a]P are first oxidized by the CYP1 enzymes to epoxides, which next are hydrolyzed by epoxide hydrolase to PAH dihydrodiols/B[a]P-7,8-dihydrodiol. A second CYP1-catalyzed oxidation at the double bond adjacent to the diol forming stereospecific PAH dihydrodiol-epoxides/B[a]P-7,8-dihydroxy-9,10-epoxide. Some of these are highly reactive electrophilic metabolites which can form stable DNA adducts or promote depurination at damaged nucleotide sites [123]. ii) In the aldo–keto reductase pathway, the PAHs are first metabolized by CYP1A1/CYP1B1 followed by epoxide hydrolase. However, here the PAH dihydrodiols/B[a]P-7,8-dihydrodiol can be further oxidized by aldo–keto reductases to a PAH dione/B[*a*]P-7,8-dione. Several human aldo–keto reductases have been implicated in this pathway, which may generate ROS and oxidative DNA damage via redox cycling of PAH *o*-quinones. iii) PAHs can also be metabolized by peroxidase reactions to reactive radical cations, which in the case of B[*a*] P will occur in the C6 position. The one-electron oxidations mediated by peroxidases or other enzymes resulting in PAH radical cations and ROS mainly result in unstable DNA adducts subjected to depurinations [124].

The AhR regulates the induction of CYP1-enzymes including CYP1A1, CYP1A2, CYP1B1 and phase II enzymes NADPH:quinone oxidoreductase (NQO1), glutathione S-transferase (GST) A2, and UDP-glucuronosyltransferase (UGT)1A1 and UGT1A6 [31]. The AhR can also directly or indirectly regulate expression of several aldo–keto reductases, together with Nrf2 [27,125]. Many of these AhR-regulated enzymes are central to the total metabolism of PAHs and directly participate in production of reactive PAH metabolites. NSCLC samples are found to express increased levels of AhR mRNA wich correlates positively with CYP1A1 expression in cases of ADC [126]. Also, polymorphisms in CYP1A1 and CYP1B1 have been linked to increased lung cancer risk [127–129]. Notably, most studies on the molecular mechanisms illustrating various steps to be involved in the carcinogenicity of PAHs were based on studies of a single compound, typically B[a]P. In real life, we are exposed to mixtures which may contain hundreds of different PAHs and other compounds likely to interfere with the metabolic activation/detoxication processes [27]. Although many factors are important determinators for the toxicity, the central role of AhR-induced upregulation of CYP1 enzymes in the bioactivation of PAHs is further illustrated by other associations found between tissue specific AhR-dependent aryl hydrocarbon hydroxylase induction/CYP1 isoforms and rates of cancer, mutagenesis, DNA adducts and toxicity of PAHs [130].

Apart from regulation of enzymes associated with PAH metabolism, the AhR also acts as a “master regulator” of numerous other genes that are linked with the process of carcinogenesis. Therefore, in addition to the regulation of formation of genotoxic PAH metabolites, activation of the AhR by PAHs can be associated with further non-genotoxic mechanisms of action of PAHs, including e.g.: perturbation of cell cycle progression, cell proliferation and programmed cell death [27], deregulation of action of hormones and/or their metabolism (including e.g. increased catabolism of steroid hormones) [131], as well as deregulation of numerous genes linked with cancer development [132]. Therefore, estimation of the AhR-activating relative potencies (REPs) (calculated relative to TCDD or to B[*a*]P as model AhR agonists) can provide an important information about toxicity/carcinogenicity of PAHs and their mixtures that are associated with PM.

A comprehensive evaluation of AhR REPs of individual PAHs, monomethylated and oxygenated PAHs has been carried out using rat hepatoma H4IIE cell line, stably transfected with a luciferase reporter gene under the control of dioxin-responsive enhancers designed as DR-CALUX assay [133]. REP values calculated relative to the TCDD-induced AhR activity, were developed for thirty abundant environmental PAHs [134], dibenzoanthracenes and benzochrysenes [106], and monomethylated chrysenes, benz[*a*]anthracenes and B[*a*]P [107–109]. Additional data have been developed also for other PAH compounds using either DR-CALUX or its variant, PAH-CALUX assays [135,136]. In general, AhR REP values expressed relative to TCDD ranging from 1 × 10^−3^ (for benzo [*k*]fluoranthene, dibenzo[*a,h*]anthracene and dibenzo[*a,k*]fluoranthene) to 1 × 10^−8^ for fluoranthene. Since various classes of AhR ligands may differentially activate human and rodent AhR, human AhR-inducing REPs have also been developed [137], using the gene reporter AZ-AhR cell line [138]. The order for REPs of individual PAHs in human cells largely corresponded with the available data from rodent-based DR-CALUX assay, although some differences up to one order of magnitude in REP values of PAHs between human and rodent cells have been observed. Higher REP values were found in human cells for some important AhR ligands among PAHs, such as indeno[1,2,3-cd]pyrene, benz[*a*]anthracene or benzo[*b*]fluoranthene, while lower REP values have been determined for methyl-substituted PAHs. The same experimental models have also been used for estimation of AhR-mediated activities of PM extracts and chromatographic fractions (non-polar and polar) of these extracts. Taken together, the AhR-mediated activity of PAHs is an important parameter for hazard/risk assessment of both PM mixtures and individual environmental PAHs, as this mode of action is highly relevant for both genotoxic and non-genotoxic effects of PAHs, as well as PAH-containing mixtures, as further discussed below.

## 6. AhR - Reactive metabolites and genotoxicity

DNA damage, mutations and genomic instability is considered a universal hallmark of all cancers including lung cancer [139]. Exogenous DNA damage may arise from cellular exposure to radiation and environmental carcinogenic compounds including PAHs from combustion PM. As the AhR regulates the induction of phase I and phase II enzymes, AhR strongly influence the formation of DNA-reactive PAH-metabolites as well as the biological stability of the parent compounds which have implication for the duration of AhR signaling. However, most mutations in human tissues are of endogenous origin. DNA damage is naturally occurring due to chemical DNA instability (e.g. depurination). It can be induced by various cellular processes including somatic recombination, endogenous reactive chemicals (e.g. aldehydes and S-adenosylmethionine), ROS and products generated as a consequence of oxidative stress (e.g. lipid peroxides) [140,141]. Because of the low contribution of exogenous agents to the mutation rate of normal cells, initiation and mutations increasing DNA instability are expected to be chiefly due to endogenous causes [140]. In fact, oxidative DNA damage is often considered to be a driver of carcinogenesis [142]. Guanine is the most frequently oxidized base. Following oxidation, it will form 8-oxo-7, 8-dihydro-guanine (8-oxoG) [143]. Due to mispairing, such lesions may result in G:C to A:T transversions during replication, one of the most common mutagenic features seen in many cancers including lung [144].

Like many other cancer types, lung cancers often have a high level of mutations in the tumor suppressor gene TP53. The TP53 gene provides instructions for making the tumor protein p53 (or p53). p53 is central in the maintenance of genomic stability, responding to DNA damage by promoting cell cycle arrest and repair, balancing transcriptional regulation of DNA repair genes and induction of apoptosis. Cells with non-functional p53 will thus accumulate more DNA damage and be more resistant to cell death. Furthermore, as the presence of TP53 mutations are found in preneoplastic lesions in the lung, it is hypothesized to be an early marker of lung cancer development [145].

Mutagenic and genotoxic effects of PM/EOM from combustion PM are well known [8,11,12]. A number of studies have shown that people exposed to combustion PM have increased levels of genotoxicity biomarkers including chromosome aberrations, micronuclei, DNA damage measured by ^32^P-postlabeling or the comet assay.

There are several approaches suggested for a rapid assessment of the carcinogenic potencies of combustion PM from various sources. These are most often based on *in vitro* assays for genotoxic/mutagenic activity of PM or EOM [22,24,146]. The mutagenic potency of EOM from a variety of combustion emissions in the Salmonella test have been found to span two orders of magnitude [24]. Chemical analysis combined with mutagenicity studies of fractionated EOM have shown that the mutagenicity is most likely due to just a few chemical classes out of which PAHs are often found to play a central role [8,9,11]. This hypothesis is further supported by studies of EOM *in vitro* which have revealed mutagenic pattern similar to that seen following exposure to PAHs [24,147], as discussed in the section below. A similar approach has been used to derive mutagenic potencies of PAHs based on mutation assay in human B-lymphoblastoid cells [148,149]. As an alternative to genotoxicity testing of PM, the levels of PAHs in organic extracts from combustion PM can be also combined with information of the specific carcinogenic or AhR potencies of PAHs based on *in vivo* and/or *in vitro* data [134,137,150]. Other carcinogenicity-linked endpoints have been also proposed to quantify relative carcinogenic potencies of PAHs [26,151] Carcinogenicity risk assessment of PAHs is often based on toxic equivalency factors (TEFs) expressed relative to B[*a*]P, based on *meta*-analysis of animal carcinogenicity studies, as proposed by Nisbet and LaGoy [150], which serve to derive carcinogenicity of mixtures of PAHs, where an individual PAH concentration is multiplied by its respective TEF [152 153]. Such approach may serve to identify principal contributors of carcinogenicity or specific toxic action of PAH mixtures, and it has suggested that cyclopenta[*c,d*]pyrene, in addition to B[*a*]P, could be a prominent contributor to the estimated mutagenicity of the PAHs found in combustion PM samples [154,155]. Similarly, dibenzo[*a,l*] pyrene and to a lesser extent benzo[*b*]fluoranthene were found to be the major contributors to mutagenic potency in extracts of DEP collected from an industrial forklift [56]. Regarding AhR REPs, specific patterns of PAH contributors to the AhR-mediated activity were identified in extracts of standard reference materials (SRM) of urban air PM (SRM 1649a), diesel exhaust particles (DEP) from heavy duty diesel engine (SRM 1650b) and DEP collected from an industrial forklift (SRM 2975) [56]. Here, the following major AhR-active compounds were identified: benzo[*k*]fluoranthene and to a lesser extent indeno[*1,2,3-cd*]pyrene, benzo[*j*]fluoranthene, dibenzo[*a,h*]anthracene in SRM1649a; benzo[*k*] fluoranthene, indenopyrene, chrysene, benzo[*b*]chrysene and benzo[*j*] fluoranthene in SRM1650b; chrysene, indenopyrene, benzo[*k*]-, benzo [*b*]-, benzo[*j*]-, and dibenzo[*b,k*]-fluoranthene and 9-methylbenz[*a*] anthracene in SRM 2975. Generally, mutagenic, AhR-mediated and carcinogenic potencies of individual PAHs seem to be independent parameters. A number of non-priority PAHs such as cyclopenta[*c,d*]pyrene, benzo[*j*]fluoranthene, benzochrysenes and methylbenz[*a*] anthracenes belong among significant AhR agonists and/or genotoxic PAHs. For example, contribution of environmental six-ring PAHs with molecular weight 302 to overall AhR-mediated activity of airborne PM and DEP is even comparable with the overall contribution of carcinogenic US EPA PAHs [156]. It is of note that potent carcinogens, such as B [*a*]P and 5-methylchrysene, may combine multiple types of toxic activities, including genotoxicity, AhR-mediated activity and tumor promotion activities (see section 9), and they occur at relatively high concentrations in polluted air.

A central role for PAHs-induced mutagenesis in human lung cancer is further substantiated by analyses of mutation spectra in various types of lung cancers. As most hotspot codons are also for the most part mutated in non-lung cancers, the location of mutations seems to be mutagen independent [157]. However, both the TP53 and KRAS mutations found in lung cancer of smokers are predominantly G:C to T:A (G to T) transversions, while other types of cancers are generally dominated by G:C to A:T (G to A) transitions including the TP53 mutations in lung cancers of never-smokers [6,81,157]. B[*a*]P is metabolically activated into BPDE which reacts with DNA predominantly at the N2-position of guanine to produce primarily N2 -guanine lesions e.g. B[*a*]P 7,8-diol-9,10-epoxide-N2-deoxyguanosine (BPDE-N2-dG) adduct. As tobacco as well as the ultimate reactive B[*a*]P metabolite BPDE most often form G to T transversions, some have argued that B[*a*]P could be the carcinogen responsible for these mutations [24,158]. Importantly, production of pro-inflammatory mediators in target tissue that is associated with PM exposure may further increase production of genotoxic B[*a*]P metabolites, including BPDE [159,160].

Importantly, several other DNA lesions are also formed after tobacco/B[*a*]P exposure. Furthermore, there are studies that have failed to find significant differences in the spectrum of mutations between smokers and never-smokers although confirming the predominance of G to T transversions in lung cancer [161]. They proposed that spectra of TP53 mutations was due to an enhanced biological selection and that smoke exposure enhanced the effects of an endogenous mutagen. G to T transversions have also been suggested to be the predominant base substitution induced by PM from urban air [147] and smoky coal (Granville et al., 2003). Other PAHs, like the highly mutagenic cyclopenta[*c,d*]pyrene, induce similar types of mutations (guanine as well as adenine transversions) as observed for B[*a*]P [162]. Furthermore, this mutation pattern may not only be reflective of PAHs, but also aromatic amines [103,163]. G to T transversions are also formed via oxidative DNA damage, including PAH *o*-quinones under redox-cycling conditions [157].

Next-generation sequencing and computational analyses have revealed very complex high-resolution mutational profiles in cancers including changes in single base substitutions, doublet base substitutions, small insertion/deletions, and copy number mutations in human cancers [164,165]. The complexity of the new data reflects the fact that the mutations are due to various endogenous factors as well as a huge number of environmental exposures, each of them resulting in a spectrum of DNA damage. Despite this complexity, there still seems to be specific mutational signatures across the spectrum of human cancer types. Each mutational signature is hypothesized to correspond to specific mutagenic processes, thus considered to help further elucidating the etiology of cancer.

The large-scale analyses allowed to comprehensively evaluate mutational spectra in various lung cancer types [165,166] as well as those induced by cigarette smoke and individual components of cigarette smoke in experimental setting [87,164,166]. Such studies have confirmed the hypothesis suggesting a role for B[*a*]P-induced mutations in lung cancer from tobacco smokers [87,164]. More specifically, a study with human pluripotent stem cells exposed to various environmentally relevant chemicals and then clonally expanded suggests that the *in vitro* high-resolution mutational signatures from B[*a*]P, dibenzo[*a, h*]anthracene, 5-methylchrysene, and dibenz[*a,j*]-acridine are similar [167]. Similarly, mutational profiles of lung epithelial cells exposed to individual tobacco smoke chemicals have confirmed and extended the previously characterized B[*a*]P mutational signatures [87]. Here, the mutational signatures arising from B[*a*]P and norharmane were both found to be similar to human lung cancer signatures attributed to tobacco smoking [87].

There is, however, no strong mutational signature seen in populations exposed to outdoor air pollution. As this may be due to a lower dose when compared to cigarette smoke as previously discussed in section 4, a central issue for lung cancer development is to explore the rate limiting steps in the development. Several approaches have been taken, as to look for sensitive endpoints for toxic responses. The low contribution of exogenous agents to the mutation rate of normal cells suggests that carcinogens including combustion PM at low doses primarily act via other pathways. Furthermore, B[*a*]P-induced gene mutations and/or chromosomal aberrations appear to be less sensitive endpoints than the initial DNA damage induced, as BPDE-dGs most often are efficiently eliminated by nucleotide excision repair [168]. However, the induced DNA damage will modulate the transcription of many genes which are predominantly involved in cell cycle regulation, apoptosis, and DNA repair [169,170]. In addition, B[*a*]P and other PAHs/PAHs-derivatives may modulate gene transcription via interactions with AhR [169], as it is further discussed in sections below.

The exception to this scenario might be a situation of sustained excessive exposure to carcinogenic agents. This seems to be the case in cigarette smokers and persons occupationally exposed to high levels of other combustion PM based on the change in mutation spectra induced, which suggest PAHs-induced mutations. It may be that the higher concentration of combustion PM/PAHs/B[*a*]P simply increases the relative mutagenic probability from B[*a*]P over that of endogenous sources for DNA damage; possibly partly as a result of impaired detoxication pathways and/or DNA repair mechanisms at higher concentrations [171].

## 7. Reactive metabolites - Cell death, inflammation and compensatory cell proliferation

PM_2.5,_ DEP, and some compounds attached to such particles may elicit formation of reactive molecules including ROS and electrophilic compounds reacting with macromolecules in various lung epithelial cells. Depending on their nature and half-life, the reactive metabolite will preferentially react with proteins or DNA giving rise to cell death, chromosomal aberrations or gene mutations [172,173]. Realizing that DNA damage from endogenous processes is probably far more prevalent than those resulting from exogenous agents [174,175], it becomes clear that processes changing the level of DNA damage by which cells will survive, enter S-phase or go into mitosis will increase the probability of accumulating gene mutation/chromosomal aberrations. Cell deaths may result in compensatory cell proliferation which is of great importance for fixation of DNA lesion, as well as an activation of ROS release in inflammatory cells which may further amplify epithelial tissue/DNA damage [176]. Accordingly, chronic tissue irritation with cell death is now regarded as an important part of lung cancer development. Importantly, the particles also contain compounds, including PAHs, which may change the level of DNA damage that the cell may tolerate and survive [177–179].

Silencing or mutation of TP53 tumor suppressor gene is considered the most prevalent oncogenic driver in lung cancer development. Genotoxic as well as various non-genotoxic mechanisms of p53 inactivation that are linked to PAHs have been reported. Repeated PM_2.5_ exposure has recently been reported to inhibit p53 expression via promoter hypermethylation [180], but p53 activity may also be more directly reduced. For a long time, it has been known that several PAHs may have so-called “stealth properties” [181,182], as they are able to covalently bind to DNA without being detected by the cells defense system. More specifically, several of the electrophilic PAH metabolites bind to DNA without triggering a proper G1-arrest. An increase in p53 can be seen, but often this p53 seems to be transcriptionally inactive as it does not lead to increased levels of p21^waf1/cip^, which are responsible for cell cycle control, blocking the transition from phase G1 to phase S. Furthermore, some PAHs are found to reduce an activation of p53 by induction of mouse double minute 2 (mdm2) protein which is a major negative regulator of p53 [183]. Reduced p53 nuclear translocation, stimulation of cell survival signals such as phosphorylation of Akt and Bad, and inhibition of DNA damage-induced apoptosis have been reported after exposure to certain PAH [177–179]. Cellular stress caused by DNA damage induces checkpoint kinase-2 (CHK2)-mediated phosphorylation and stabilization of the E2F1 transcription factor. The activation of a subset of pro-apoptotic E2F1 target genes, including apoptotic peptidase activating factor 1 (APAF1/Apaf1) and tumor protein 73 (TP73/p73) leading to apoptosis is attenuated by AhR-binding to E2F1 [184].

Importantly, B[*a*]P itself (as well as other PAHs) forms numerous metabolites with poorly characterized toxicological profiles, which might further modulate cellular responses to DNA damage [27]. Some of these PAHs have also been reported to have AhR-dependent activity linked to the regulation of cell proliferation, differentiation, senescence and programmed cell death [185]. The link between AhR-signaling and control of cell growth and proliferation is complex and may depend on cell phenotype as further discussed in section 9. Weak mitogenic activity which may also occur via increased intracellular calcium concentrations [Ca^2+^]*i* activation of EGFR and insulin receptor signaling, or estrogen receptors (ER) [186–188], elicited either by parent PAHs or their metabolites. Furthermore, an AhR-dependent disruption of contact inhibition induced by PAHs has been reported for a number of AhR-activating PAHs, probably linked to induction of JunD/cyclin A pathway [189].

Chemicals interfering with the cellular defense system, giving anti-apoptotic or mitotic signaling, would change the balance between cell death, cell survival and cell proliferation following endogenous DNA damaging events. If not compensated with increased DNA repair, it is likely that the result would increase the probability of permanent genetic damage. This hypothesis is supported by the fact that low doses of combustion PM/PM_2.5_ mostly result in cancers with “natural” mutations, in line with important roles also for the non-genotoxic properties of PAHs in lung cancer development.

## 8. Intracellular Ca^2+^-signaling, non-classical genomic and non-genomic AhR-pathways

While the classical or canonical genomic AhR-pathway leading to activation of CYP1A1/−1A2 and CYP1B1 through dimerization with Arnt is clearly essential for the formation of mutagenic metabolites and oxidative stress responses from PAHs in combustion PM, it cannot explain all effects observed from AhR ligands [190]. Non-classical or non-canonical effects involve alternative genomic pathways where AhR interacts with other transcription factors, such as the estrogen receptors (ERs) or the RelA and RelB subunits of the nuclear factor-κB (NF-κB), and which regulates a number of other genes, independently of a canonical XRE/DRE (xenobiotic or dioxin response elements) binding [191–193]. In addition, AhR may also function as signaling molecule in the cytosol controlling activation of c-Src and calcium (Ca^2+^) signaling through the so-called non-genomic pathway [190,192]. These non-classical pathways enable regulation of several processes relevant for carcinogenesis and tumor development, including inflammation, cell-to-cell communication, cell growth and proliferation, and cell migration which is discussed in more detail in the sections to follow (Fig. 1).

The NF-κB family of transcription factors are key regulators of inflammatory responses, including a number of cytokines, chemokines, and adhesion molecules which play central roles in cancer development [176,194]. Extensive crosstalk between AhR and NF-κB has been reported [195–197]. TCDD exposure and AhR overexpression increased NF-κB activity and IL-6 expression in lung cells [198]. TCDD also induced dimerization of AhR and RelB of the alternative NF-κB pathway and up-regulation of CXCL8 through a novel RelB/AhR response element (RelBAHRE) in macrophages and breast cancer cells [196,199]. Furthermore, B[*a*]P may induce CXCL8 expression in primary human lung macrophages through binding of AhR to consensus XRE sites in the CXCL8 promoter, and B[*a*]P administration increased pulmonary inflammation in mice [200]. AhR can also dimerize with the p65-submunit of NF-κB and activate κB-sites in the IL-6 and c-myc promoters [198,201]. However, AhR-deficient mice have been reported to display elevated NF-κB activity and inflammation in the lungs after inhalation of lipopolysaccharide (LPS), cigarette smoke, or crystalline silica [202,203]. AhR knockout has been also shown to increase inflammatory signaling in lung adenocarcinoma A549 cells [204]. Furthermore, AhR activation may suppress pulmonary inflammation induced by crystalline silica [203]. The receptor therefore seems to elicit both pro- and anti-inflammatory functions through enhancement and suppression of NF-κB activity in the lung and other tissues. A study in human bronchial BEAS-2B cells shows that this dual action may occur even within the same cell type. Both constitutive and ligand-activated AhR elicited a weak to moderate pro-inflammatory signal increasing CXCL8 and CCL5 release but seemed to suppress p65 activation and chemokine responses in combination with stronger activators of the classical NF-κB pathway, such as polyinosinic:polycytidylic acid (Poly I: C) or tumor necrosis factor (TNF)-α [205]. The interaction of AhR with members of the NF-kB family is an important aspect, as unresolved chronic inflammation is considered to be an important hallmark of cancer [194].

While non-activated AhR in its resting state is often depicted as “freely floating” in the cytosol, some studies suggest that at least a fraction of the AhR is anchored to the cell membrane, most likely in close connection with cholesterol rich regions such as the caveolae. AhR appears to bind directly to caveolin-1 (Cav1), and this binding is affected by exposure to AhR ligands [206,207]. A close connection between AhR and the cell membrane makes sense, as most AhR ligands are highly lipophilic and thereby distribute within the phospholipid bilayer, rather than dissolving into the aqueous cytosol [208,209]. Caveolae are believed to be central in the uptake of lipids and lipophilic compounds [210,211]. In line with this, polychlorinated biphenyls (PCBs) have been shown to accumulate in caveolae [212], suggesting that AhR is located at the regions where its ligand occur at the highest concentrations. This also places AhR in close contact with major cell signaling components, since a variety of different receptors and ion-channels cluster in cholesterol-rich micro domains. Studies in human microvascular endothelial cells suggest that pyrene and PAH-rich DEP-derived EOM trigger AhR-dependent Ca^2+^-signaling, possibly through activation of transient receptor potential canonical (TRPC) channels [213,214]. This response occurred rapidly after approximately two min of exposure, preceding transcriptional regulation. Similarly, DEP-EOM and phenanthrene were reported to stimulate Ca^2+^-influx and membrane depolarization in airway sensory nerve fibers from guinea pigs through AhR-dependent activation of TRPA ion channels [215]. AhR-mediated Ca^2+^-signaling through the so-called non-genomic pathway seems to be a central step in the regulation of TCDD induced cyclooxygenase 2 (COX-2) activation, prostaglandin release and inflammation [190]. Dysregulation of Ca^2+^-signaling is frequent in many cancer types and has been linked to tumor progression. Furthermore, aberrant expression of TRP-channel such as TRPC and TRPM has been reported in lung cancer and other cancer types and has been linked to EMT, cell proliferation, invasion and promotion of cell survival and suppression of apoptosis [216,217]. Importantly, these effects have been described for pyrene and phenanthrene, PAHs that traditionally have been considered weak AhR activators due to limited effects on classical AhR:Arnt signaling [150]. Although the potential role of AhR-induced Ca^2+^-responses in lung cancer development remains to be clarified, this underscores that models developed to assess AhR REP based on XRE/DRE driven reporters may not account for the non-classical effects of AhR ligands.

It should also be considered that PAHs may further activate intracellular Ca^2+^-signaling not only through AhR-driven responses. Beta-adrenergic receptors (β-ARs) have been detected in cancer cells of the breast, prostate, and skin as well as in lung cancer [218,219]. Numerous studies have linked this receptor to a variety of cellular phenomena such as cell proliferation and motility, cell apoptosis resistance, EMT, metastasis, and angiogenesis. Some constituents of tobacco smoke (e.g. 4-methylnitrosamino-1-(3-pyridyl)-1-butanone, a derivative of nicotine) are known agonists of β-ARs [220,221], and may regulate tumor cell proliferation and migration which are inhibited by beta-blockers (e.g. propranolol). Interestingly, β-AR, especially β2-AR, is also associated to the intracellular Ca^2+^ increase induced by B[*a*]P. Indeed, Mayati and coworkers demonstrated using an endothelial cell model that B[*a*]P induced intracellular calcium concentration through binding to β2-AR, and activation of G protein/adenylyl cyclase/cAMP/EPAC/phospholipase C pathway [222]. This effect was also inhibited by beta-blockers. Besides, β-AR pathway can modulate lung cancer cell resistance, and some works indicate that beta-blockers can slow down the onset of therapeutics resistance especially those associated or interacting with EGFR [223]. Although there is no consensus on the effects of betablocker treatment, it is interesting to note the role of β-ARs in lung cancer primarily have been linked to ADC- and EGFR-driven mutations, as reviewed elsewhere [219,224].

Another central part of AhR non-genomic signaling is the rapid c-Src-mediated activation of EGFR [225–227]. EGFR appears to regulate cytokine responses in DEP-exposed bronchial epithelial cells [228] and it may contribute to the AhR-induced inflammatory responses. The AhR-dependent activation of c-Src has also been found to be important in the TCDD-mediated regulation of COX-2 and prostaglandins [229]. COX-2 is known to be a key enzyme producing prostaglandins which may contribute to tumorigenesis including lung cancer [230–232]. Importantly, different ligands induce different responses upon AhR activation, also in the case of EGFR-mediated effects. A recent study revealed that in contrast to dioxin-like chemicals, the treatment of human epithelial cells with PAHs including B[*a*]P results in an auto-/paracrine activation of EGFR, which can be an important contributing factor in AhR-mediated tumor promotion [233]. AhR-induced activation of EGFR may also occur in concert with traditional genomic signaling and may induce cancer cell proliferation [116,234], and has also been reported to cause resistance towards EGFR tyrosin kinase inhibitor (EGFR-TKI) treatment of adenocarcinoma through Src-mediated non-genomic signaling [115]. Similar to AhR, EGFR may localize in the caveolae and interact with Cav1. Downregulation of Cav1 has been reported to enhance sensitivity towards EGFR-TKIs in lung adenocarcinoma cells (PC9) harboring EGFR mutations [235]. Both AhR overexpression and exposure to the AhR ligand PCB77 appear to increase Cav1 levels in caveolae [207,236]. Thus, the role of AhR in regulation EGFR activation and EGFR-TKI sensitivity, likely involves both c-Src and Cav1. As reviewed elsewhere, Cav1 has also been implicated in multiple stages of lung cancer development, including cell proliferation, migration, apoptosis and drug resistance [237]. Hence, the importance of AhR-Cav1 crosstalk likely extends beyond regulation of EGFR and warrants further studies into the role of non-genomic AhR signaling in ordered membrane microdomains for development of lung cancer. Collectively, these findings point towards a potential role of AhR in air pollution-mediated lung ADCs with EGFR driven mutations and lung cancer.

The pattern of AhR signaling with both genomic and non-genomic pathways and localization of at least a pool of cellular AhR at the caveolae interacting with Cav1, strongly resembles steroid receptor signaling pathways. Also, a pool of the estrogen, androgen, progesterone and glucocorticoid receptors (ER, AR, PR and GR) interact with Cav1 and signal through non-genomic pathways, in addition to their classical genomic pathways, in a pattern similar to AhR, involving both rapid c-Src and calcium responses. As reviewed elsewhere, these non-genomic steroid receptors signaling pathways appear important in cancer development, especially in estrogen and androgen sensitive cancers such as breast and prostate cancers. [238,239]. Due to the many shared features, it seems reasonable to expect that crosstalk between AhR and ER/AR non-genomic signaling may occur. More specifically, the interactions between AhR and the genomic signaling of steroid receptors are well known and include interference with ER, AR, PR and GR, although the crosstalk with ER is by far the best described. AhR can interfere with ER signaling through several mechanisms including induction of CYP1A1/1B1 which can metabolize estrogen, thereby reducing intracellular estrogen concentrations and ER activation, AhR: Arnt-mediated suppression of transcriptional activity of ER (“squelching”), and direct interactions leading to AhR:ER dimerization. However, AhR may both suppress and induce ER-regulated genes [191]. As reported for AhR, there also seems to be a crosstalk between ER and EGFR signaling in lung ADC. ERα (but not ERβ) appears to be highly correlated with presence of EGFR mutations in lung ADCs of female never-smokers [240]. The EGFR driver mutations observed in air-pollution associated ADC in never-smokers, were also far more frequent in women [38] which also appear more likely to develop ADC than SCC and to have a higher risk of developing lung cancer from smoking, compared to men [32]. However, while these observations are compatible with the involvement of sex steroid hormones, the interaction between PAHs and ER in lung cancer development remains elusive, and AhR-ER crosstalk has so far not been explored in lung cells with EGFR driver mutations.

Besides the presence of AhR at the plasma membrane, previous works have also pointed to the existence of a pool of AhR located in mitochondria, with possible consequences in terms of the metabolic reprogramming involved in tumor development. Thus, AhR has been shown to interact with one sub-unit of the mitochondrial F0F1-ATPase, namely the ATP5a1, in several cell lines (hepatic cells, lymphoma cells) [241]. Interestingly, the authors demonstrated that upon activation of AhR by TCDD, the AHR:ATP5α1 interaction was disrupted and a mitochondrial hyperpolarization occurred in an AhR-dependent and transcription-independent manner. It is noteworthy that under such conditions, a decrease in ATP production was also observed, although not significant. This led the authors to propose a role in the regulation of mitochondrial metabolism for this so-called «mito-AhR» which was shown to be located in the inter-membrane space of the organelle in Hepa1c1c7 cells [242]. Interestingly, Lagadic-Gossmann and coworkers previously showed in the epithelial hepatic cell line F258 that B[*a*]P was capable not only to induce a mitochondrial hyperpolarization [243], but also to trigger a glycolytic reprogramming [243], both being involved in survival signals supporting tumorigenesis [244]. Metabolic reprogramming is one of the hallmarks of development of lung cancer and other tumors [245,246] and recent data suggest that enhanced glycolysis may be central in PM_2.5_ induced NSCLC [247]. Intriguingly, DEP has also been reported to induce mitochondrial hyperpolarization in primary human T-cells [248] and PM_2.5_ has been reported to suppress mitochondrial-driven apoptosis through AhR dependent mechanisms [249]. Collectively this suggests that the role of the mitochondrial pool of AhR in lung cancer could be worth exploring. Furthermore, as cancer-related metabolic reprogramming can rely on changes in pH homeostasis [250] and as B[*a*]P is capable of eliciting changes in intracellular pH [251], it would also be interesting to test a role for such pH modifications. In line with this, note that calcineurin homologous protein isoform 2 (CHP2) was described to support tumor survival in non-small cell lung cancer, via the sodium/hydrogen exchanger (Na^+^/H^+^ exchanger, NHE) isoform 1 [252], *i.e*. an important transmembrane pH regulator that we showed to be activated by carcinogenic PAHs, including B[*a*]P [253]. Another important player worth investigating in this network would be the ATPase inhibitory factor 1 (IF1), that is, the physiological inhibitor of the F0F1-ATPase. Indeed, the activity of this peptide is sensitive to pH variations and has been linked to metabolic reprogramming and tumorigenesis [252,254]. Its gene expression seems to be modulated upon PAH exposure via AhR as well as β2-AR [255,256]. With respect to that, a previous paper has found IF1 as a target for PM_2.5_, possibly related to immune and inflammatory responses in pulmonary fibrosis [257].

## 9. Cancer promotion including cell-to-cell communication, EGFR activity, extracellular vesicles and miRNA

### 9.1. Disturbance of cell-to-cell junctions and contact inhibition

Disruption of intercellular communication mediated via various types of cell-to-cell junctions, including gap junctions (GJs), adherens junctions (AJs) or tight junctions (TJs), and associated deregulation of cell adhesion are important mechanisms linked with cancer development and cancer promotion. The GJs, which connect neighboring cells allow continuous exchange of small molecules, and thus contribute the maintenance of tissue homeostasis, proliferation control and regulation of epithelial cell polarity, which makes them important players also in lung tumorigenesis [258,259]. It has been reported that connexins have tumor suppressive roles in lung tissue [260,261]. Overall, both connexin proteins themselves and GJs (which they form) play a major role in cancer development and progression [262].

The down-regulation of gap junctional intercellular communication (GJIC) that is facilitated by GJs via the action of tumor promoting compounds, can contribute to the removal of an initiated cell from the growth suppression of neighboring cells, and it may thus serve as a marker of tumor promotion [263–265]. A number of carcinogenic chemicals have been observed to down-regulate GJIC and/or connexin expression in cell models derived from various tissues, including the lungs. The shortlist of potential tumor promoters acting via GJIC inhibition also includes PAHs, in particular those with low molecular weight that are associated with PM, but primarily are present in gas phase of polluted air. Several low molecular weight PAHs (including both parent PAH compounds and methylated PAH derivatives) have been demonstrated to inhibit GJIC in rat liver cell lines [266–268]. This toxic mode of action of PAHs might be inversely related with their ability to activate the AhR as illustrated for methylated benz[*a*]anthracenes [107]. Down-regulation of GJIC has also been observed for complex mixtures of PAHs, including cigarette smoke, cigarette smoke condensate or extracts of DEP [269–271].

Although PAHs and their impact on GJIC have been studied mostly in the context of liver tissue, several studies have also addressed their impact on cell models derived from lung epithelium. Lung alveolar epithelial cells express several connexin species, proteins, which couple cells via formation of GJs [272]. In murine C10 lung cells, a non-tumorigenic type II alveolar pneumocyte and progenitor cell type of lung adenocarcinoma, 1-methylanthracene, a well-known GJIC inhibitor, has been shown to block GJIC, activate ERK1/2 and to induce expression of pro-inflammatory regulators [273]. PAHs, such as fluoranthene and B[*a*]P may also interact to elicit genotoxic effects, GJIC inhibition and up-regulation of inflammatory mediators in this lung cell model [274]. In human bronchial epithelial HBE1 cells, low molecular weight PAHs have been reported to inhibit GJIC [275], again confirming that this mode of action is not limited to liver cells.

At present, most of the reported effects of PAHs on GJs and GJIC appear to be AhR-independent. Nevertheless, inhibition of GJIC seems to be connected also with AhR-regulated disruption of cell adhesion and cell proliferation control, which will be further outlined below. More-over, inflammation is known to modulate effects of PAHs on GJIC and related endpoints [276,277]. The exposure to PAHs is a part of complex effects of PM on lung tissue, which include induction of oxidative stress and inflammation. It is likely that a combination of these effects will lead to suppression of GJIC in alveolar and/or bronchial epithelium during PM exposure, thus contributing to promoting effects of PM and associated PAHs.

AhR activity has also been reported to contribute to alterations of AJs and cell adhesion [278,279]. Exposure to PAHs or their mixtures have been linked with down-regulation of E-cadherin, which is a principal constituent of AJs. The disruption of cell-to-cell junctions mediated by E-cadherin and their homeostatic functions may lead to deregulation of cell proliferation in target cells. Notably, AhR has been shown to play an active role in proliferation control in lung adenocarcinoma cells [280,281]. Furthermore, PAHs have been documented to exhibit tumor-promoting properties in cell transformation assay *in vitro* [282]. PAHs have been found to inhibit growth suppressive mechanisms such as contact inhibition, leading to an AhR-dependent enhanced cell proliferation [189,283]. In several liver cell models, activation of the AhR leads to disruption of contact inhibition, as well as to deregulation of proteins forming AJs and participating in intracellular signaling. PAHs acting as AhR ligands can alter cell proliferation control leading to disruption of contact inhibition and to down-regulate GJIC via enhanced Cx43 degradation in rat liver epithelial cells [284]. The AhR-mediated disruption of contact inhibition and increased cell proliferation are linked with disruption of Wnt/β-catenin signaling as well as down-regulation of E-cadherin [285,286]. Together, these data suggest a connection between disruption of growth suppression via deregulation of contact inhibition and removal of cells from the growth suppression of neighboring cells, which is paralleled by GJIC inhibition and down-regulation of other types of cell-to-cell junctions.

In addition to their impact on GJs and AJs, PM or PAH exposure can also affect tight junction proteins and disrupt the integrity of lung TJs, which are important for formation of epithelial barrier, preventing access of inhaled material to sub-epithelial layers [287]. Inflammation, which plays a key role in the development of lung diseases, leads to deregulation of TJ functions and their constituents, which can be also associated with induction of EMT [288,289], as discussed further on. Disruption of lung TJs may contribute to increased susceptibility to lung diseases and promote inflammatory responses within lung tissue. PM components have been shown to disrupt TJs and deregulate expression of TJ proteins within lung or bronchial epithelium [290]. Their effects could be linked to induction of pro-inflammatory cytokines, such as IL-6 and generation of oxidative stress [291]. Exposure to combustion particles may also result in disruption of epithelial barrier integrity, as evidenced e.g. for DEP exposure [292] or during exposure to wood smoke [293]. Regarding the effects of individual PAHs, B[*a*]P has been reported to disrupt barrier in endothelial cells, without directly affecting expression of TJ proteins [294]. These results again confirm that PAHs or their complex mixtures may affect multiple types of cell-to-cell junctions, and that at least some of these effects are dependent on the AhR activation.

Activation of the AhR has been reported to activate numerous signaling pathways that are associated with both the deregulation of inflammatory responses and simultaneous regulation of epithelial cell phenotype, including cell-to-cell junctions. Non-canonical genomic AhR-signaling involves crosstalk with several other transcription factors and signaling molecules independently of Arnt activation [193]. As previously discussed, AhR ligands may also act through non-genomic AhR-signaling where AhR functions as a signaling molecule in the cytosol, regulating c-Src non-receptor tyrosine kinase and Ca^2+^ signaling, and affecting ordered lipid domains within cell membranes [190,295], thus providing a direct link between cell junction protein complexes and membrane structure. Activation of c-Src, often linked also with an increased activity of MAP kinases, can indeed impact both structural and signaling functions of cell-to-cell junctions, including GJs, AJs or TJs, but it has been also implicated in TCDD-mediated upregulation of COX-2 [229], a key enzyme producing prostaglandins which may contribute to tumorigenesis including lung cancer [231,232]. As mentioned above, the study of Vogeley et al. [233] also revealed that the treatment with PAHs such as B[*a*]P results in an auto-/paracrine activation of EGFR, which could be another contributing factor in AhR-mediated tumor promotion.

### 9.2. EGFR-mediated tumor promotion

The receptor tyrosine kinase EGFR regulates the activity of pro-oncogenic pathways including the mitogen activated protein kinases (MAPKs) ERK1/2 and mammalian target of rapamycin (mTOR), which both promote cancer cell proliferation. Mutations such as the L858R mutations in exon 21, or deletions in exon 19 may lead to overactivation of the EGFR enhancing the stimulation of cell proliferation [296]. Furthermore, several studies suggest that AhR may regulate EGFR activation, through a non-genomic pathway involving c-Src [226,227,233,234]. Thus, modulation of the EGFR activity via PAHs and other AhR ligands could be a contributing factor to cancer cell proliferation and tumor promotion.

As previously discussed, ambient air PM_2.5_ appears to stimulate tumor promotion of cells harboring EGFR driver-mutations, such as the L858R mutation [38]. Several studies suggest that AhR could be involved in this process. Nuclear localization of AhR has been reported to be more common in lung cancer from women, non-smokers, adenocarcinoma and NSCLC patients with the EGFR exon 19 (E746–750A) deletion [114]. High AhR expression has also been reported from adenocarcinoma cell lines and in human ADC biopsies AhR immunostaining was higher than in normal bronchial tissue and SCC [297]. By contrast, AhR appears to suppress KRAS-driven lung tumor formation [117], which is more common in smokers than never-smokers [6]. As such, it appears that the role of AhR in lung tumor promotion may be more restricted to ADC with EGFR driver-mutations. AhR may also strengthen the resistance towards EGFR tyrosine kinase inhibitor (EGFR-TKI) in NSCLCs through non-genomic Src signaling [115,116]. Intriguingly, cancer cells appear to utilize this AhR-mediated pathway. Cancer-associated fibroblast (CAFs) have been reported to stimulate AhR-dependent proliferation and EGFR-TKI resistance in NSCLCs through production and release of the tryptophane metabolite and potent AhR ligand kynurenine [116]. The kynurenine-AhR axis is dysregulated in a number of cancers and has been associated not only with increased cell proliferation, but also immune evasion, neo-angiogenesis, metastasis, and chemoresistance [298]. Moreover, kynurenine from tumor-repopulating T-cells (TRCs) have been reported to drive AhR dependent upregulation of programmed cell death protein 1 (PD-1) in CD8^+^ T cells, with potential consequences for cancer immunotherapies [299]. PD-1 inhibits immune responses and promotes self-tolerance by modulating T-cell activity which may contribute to immune evasion [300]. This AhR-kynurenine-PD-1 pathway may also be activated in air pollution induced lung cancer. PM_2.5_, cigarette smoke and B[*a*]P has been shown to induce PD-1 ligand (PD-L1) in lung epithelial cells and macrophages, and the therapeutic effects of anti-PD-L1 antibody treatment (pembrolizumab) appear to be limited to lung cancers with high AhR expression levels, in both patients and mouse models [38,301].

In extension of the above, Wang et al [302] recently reported that long-term PM_2.5_ exposure [90 days) induced persistent activation of EGFR, cell proliferation, anchorage-independent growth, and tumor growth (xenograft mouse model) in human adenocarcinoma NCI-H1975 cells which harbors both the EGFR L858R and T790M mutations. Induction of proliferation and anchorage-independent growth was also observed in human lung cancer PC9 cells which carry a Glu746-Ala750 deletion mutation in exon 19 of the EGFR gene, while in human A549 lung cancer cells with KRAS-mutations but wild-type EGFR PM_2.5_ exposure only induced a transient EGFR activation and a nonsignificant increase in anchorage-independent growth [302]. In H1975 cells, the exposure to PM_2.5_ induced approximately 5-fold increase in colony formation ability, but in PC9 and A549 cells PM_2.5_ exposure caused a less than 2-fold increase [302]. These data suggest that PM_2.5_ stimulates EGFR activation and cell proliferation in a variety of lung cancer cell lines, but the responses were considerably enhanced in cells harboring both the L858R and T790M mutations [302], which both are common in ADC from never smokers [38].

PM_2.5_-exposure has also been shown to induce an AhR-dependent transcriptional activation of transmembrane serine protease 2 (TMPRSS2) and subsequent expression of the IL-1 family member IL-18 that may promote cancer progression. AhR nuclear expression also correlated with TMPRSS2 and IL18 expression and cancer stage in human lung cancer tissue [302]. Although the link between the AhR-TMPRSS2-IL18 pathway and EGFR activation was not specifically explored, the study provides a potential link between AhR activation and ADC with EGFR driver mutations. On the other hand, AhR expression has been reported to suppress lung cancer metastasis after orthotopic implantation of human adenocarcinoma cell lines (H1975, A549 and H1299) in SCID CB.17 mice, suggesting that AhR suppresses lung carcinogenesis irrespective of the dominant oncogenic driver [303]. Low AhR expression levels were also associated with faster cancer progression and reduced survival in lung ADC patients [303]. A likely explanation for this apparent contradiction could be thedifferences in effects of constitutive AhR activity versus PAH-induced AhR activation. There is a considerable diversity in AhR-regulated responses induced by different ligands [191], and the native AhR in unstimulated cells appears to affect the regulation of different gene clusters than those regulated upon ligand activation [304]. Importantly, while PAH exposure activated EGFR, this was not the case for dioxins, which underscores the variability in effects induced by different ligands [233].

Based on the above, we suggest that AhR may induce proliferation of lung cancer cells through mechanisms involving both non-genomic activation of EGFR, and genomic activation of NF-κB and its target genes such as TMPRSS2, leading to inflammatory responses regulated by members of the IL-1 cytokine family, such as IL-1β and IL-18. It seems that these effects may be enhanced in EGFR-driven adenocarcinoma, especially by the L858R mutation in exon 21, and that the involvement of AhR is restricted to PAH-mediated activation, since dioxins may not activate EGFR to a similar extent and unliganded constitutive AhR appears to suppress lung tumor progression independent of the driver mutation.

### 9.3. Extracellular vesicles and miRNA

Extracellular vesicles (EVs) are nanostructures produced by all cells, mediating cell-to-cell communication by exchanging proteins, nucleic acids and lipids or organelles (e.g. mitochondria) [305–307]. They constitute a heterogeneous population including exosomes (Exo; less than 200 nm), microvesicles (MV; 100–1000 nm) and apoptotic bodies. EVs are detected in various biological fluids [308], and suggested to participate in the maintenance of cellular homeostasis and intercellular communication including immune responses, cell proliferation, tissue repair and angiogenesis. EVs contribute to inflammation by containing cytokines, accordingly EVs containing high concentrations of biologically active TNF-α produced by alveolar macrophages was detected in bronchoalveolar lavage fluids (BALFs) during lung injury [309,310]. Damaged epithelial cells may also produce EVs that recruited pro-inflammatory M1 macrophages [311]. These nanostructures are also suggested to contribute to the growth and worsening of cancers. EVs produced by lung cancer cells are reported to stimulate the production of the pro-angiogenic factor vascular endothelial growth factor (VEGF) and increase vascular permeability and extracellular matrix remodeling [312]. Furthermore, an increase in EVs containing cell death protein ligand-1 (PD-L1) suggested to be involved in tumor immune evasion observed in patients with non-small cell lung cancers, who were non-responders to treatment [313]. Notably, PD-L1 is known to be under the control of AhR [301].

An increasing amount of evidence suggests that environmental pollutants can modify the production of EVs, and that they are involved in the appearance or progression of diseases linked to environmental exposures including lung cancer [314]. Tobacco-smoke, PM_2.5_ and PAHs have been shown to trigger EV release from different lung cell types (macrophages, bronchial epithelial cells, endothelial cells, platelets) [315]. PAHs such as B[*a*]P, dibenz[*a,h*]anthracene, or benz[*a*]anthracene, have been shown to increase EVs production by endothelial cells [316]. However, until now only limited data exist concerning the role of AhR in EVs production and content, especially upon exposure to air pollutants such as tobacco-smoke, PM_2.5_ or PAHs. Recently, it was demonstrated, using endothelial and hepatic cell models, that PAHs such as B[*a*]P may increase exosome production through AhR activation [316,317]. The inhibitory effect of naringenin (a flavonoid targeting AhR pathway) on EV production in BEAS-2B cells exposed to cigarette smoke extract could indicate a role for AhR also in lung epithelial cells [318]. Furthermore, pyrene, a weak agonist of canonical AhR signaling but potent inducer of AhR non-genomic Ca^2+^ signaling [214], increased exosome production using constitutive androstane receptor (CAR) pathway [317].

EVs may also contain miRNAs/ncRNA, a class of RNAs that regulate gene expression by interacting with their target mRNAs to induce their silencing, thereby influencing the cell response [319]. Via regulation of oncogenes or tumor suppressors, miRNAs can modulate tumor formation and contribute to lung cancer development [320–323]. Interestingly, several experimental and epidemiological studies report that exposure to various sources of combustion PM such as DEP, industrial/biomass combustion and cigarette smoking alter miRNA levels [324]. For example, DEP exposure in human lung cells upregulated miR-21 which has previously been identified as an ‘oncomir’ candidate by targeting cell proliferation and EMT through regulation of the PTEN/AKT signaling pathway [325]. Furthermore, loss of miR-29a is associated with cdc7 kinase accumulation and has been suggested as a mechanism to acquire resistance to cigarette smoke-induced DNA damage allowing the cells to proliferate [326].

The miRNAs have also been studied as biomarkers of interest in lung cancer [327] as diagnostic and/or prognostic tools [328,329]. More recently, EV-derived from biological fluids and their miRNAs have been proposed as a potential source of biomarkers for exposure and effects of environmental pollutants. Changes in extracellular miRNAs have been correlated to different sources of PM including DEP [330,331], traffic-related air pollution [332] and cigarette smoke [333–336]. Some miRNAs are commonly deregulated in lung cancers and as a result of exposure to air pollution, and they have been suggested as interesting biomarkers for the detection of sensitive human populations [337]. Furthermore, miRNAs following exposure to cigarette smoke are also suggested to contribute to a modification of the tumor microenvironment towards a pro-inflammatory response [338] and to be pro-angiogenic [339,340].

Finally, an increasing number of studies have shown that some miRNAs target AhR and *vice versa*, that AhR regulate miRNAs following oncogenic changes induced by PAHs [341,342]. In fact, AhR has been proposed as a key regulator in controlling miRNA levels in lung [343]. Accordingly, without activation, AhR suppressed the expression of the cancer-associated miR-96, whereas chronic cigarette smoke markedly increased its level by a mechanism independent of classic AhR activation by ligands [343]. Such ligand-independent regulation of miR-196a by AhR has been described by Hetch et al. [344] in lung fibroblasts controlling their apoptosis and potentially regulating the hallmarks of cancer as previously suggested [345]. By contrast, we and others recently reported the ligand-dependent AhR activation of miR-132 expression in blood cells [346,347]. This miRNA may possess pro- or anti-tumor functions depending on cancer [348]. Altogether, these elements reveal the interest and the complexity of miRNAs in air pollution-induced lung cancers and underline the need to further explore biological importance of the AhR in miRNA-induced processes, notably in link with EVs.

## 10. Role of PAHs and AhR in regulating the tumor microenvironment (TME)

### 10.1. Tumor microenvironment - Immune cells and stromal cells

Tumor cells are surrounded by non-malignant stromal cells which play a critical role for the survival, growth, progression, and metastasis of cancer cells. It is important to note that the development of metastasis is the cause of more than 90% of cancer mortality, and that the metastasis of tumor cells depends on the support of their microenvironment. Non-malignant stromal cells are a heterogeneous cell population forming the structure of the tumor microenvironment (TME) and include cancer associated fibroblasts (CAFs), endothelial cells, adipocytes and pericytes. Interestingly, a recent study showed that elevation of the protein fibroblast growth factor 2 (FGF-2) expression involves AhR signaling resulting in pericyte proliferation in the TME. Consequently, increased FGF-2 signaling and proliferation of pericytes leads to accumulation of tumor associated macrophages (TAMs) and metastasis [349].

Moreover, infiltrating adaptive and innate immune cells play a critical role in the TME and exert an anti- or pro-tumorigenic effect on the development of cancer. For instance, regulatory B cells producing IL-10 may contribute to immunosuppression in the tumor microenvironment. Regulatory B cells differentiation is promoted by the key tryptophan metabolite L-kynurenine (L-Kyn) in an indoleamine 2,3-dioxygenase (IDO) and AhR-dependent mechanism [350]. In addition to B cells, recent studies have shown that AhR activation by TCDD leads to accumulation of tumor associated myeloid cells (TAMCs) including myeloid derived suppressor cells (MDSCs) or TAMs [351]. The importance of immunosuppressive TAMCs and the central role of the TME has been demonstrated for the progression and metastasis of various malignancies including lung and breast cancer [352,353].

Furthermore, recent reports have shown a critical role of AhR in the recruitment of MDSCs and TAMs in adipose tissue of TCDD-treated mice [354] and during the development of glioblastoma [355,356]. The AhR has been found to induce the expression of immunoregulatory enzymes and factors such as arginase 1 (Arg1], IDO, IL-10 and the S100 calcium binding protein S100A9 which are important for the immunosuppressive function of TAMCs by creating a tumor-promoting microenvironment [357–360]. Additionally, cytokines, chemokines, and growth factors are soluble factors and important components of the TME since they regulate the recruitment and migration of immune cells as well as tumor cells [361]. The important role of IL-1β has also been demonstrated in AhR-mediated (TCDD-induced) development of lymphoma [96]. These studies indicate that IL-1β signaling creates a tumor-promoting microenvironment contributing to tumor growth and metastasis as reported previously [362,363]. Additionally, numerous studies confirmed the AhR-dependent upregulation of IL-1β in macrophages and other cell types after treatment with PM, PAHs and TCDD [352,353].

In summary, the literature supports the conclusion that activation of AhR generates a pro-tumorigenic microenvironment that tumors evolve to escape the immune response, enabling progressive tumor growth and metastasis. Consequently, the AhR may play a critical role in the TME of various cancer types by modulating the recruitment and function of infiltrating immune cells. Because AhR can be regulated by small molecules, the AhR has been suggested to be an attractive target for the tumor microenvironment and immunotherapy to treat cancer [113,364,365].

### 10.2. Angiogenesis and tumor growth

Formation of new blood vessels, neo-angiogenesis, is an essential part of tumor development in lung cancer and other cancers [366]. Development of different AhR knockout mouse models in the 1990 s revealed that AhR deficiency caused cardiac hypertrophy, vascular abnormalities in multiple organs and altered blood pressure [367]. These studies pointed towards a central role of AhR in angiogenesis. The central role of AhR cardiovascular development and homeostasis has been extensively reviewed elsewhere [367–370] and will therefore not be discussed in detail here. Among the angiogenic factors affected by AhR activation is the vascular endothelial growth factor (VEGF), which is a key regulator of angiogenesis.

*In vitro* exposure of a coculture of eosinophilic (EoL-1) cells and human umbilical vein endothelial cells (HUVECs) to B[*a*]P, was reported to promote HUVEC growth through ERK1/2 mediated VEGF expression and release from the EoL-1 cells [371]. Similarly, benzyl butyl phtalate induced VEGF release, stimulation angiogenesis *in vitro* and *in vivo* through AhR non-genomic activation of ERK1/2 in hepatocarcinoma (Huh7) cells [372]. AhR has been reported to induce VEGF expression in HepG2 cells through activating transcription factor 4 (ATF4), which may be under regulation of the ERK172 pathway [373]. Thus, the angiogenic VEGF-signal appears to arise from activation of AhR in both immune cells and cancer cells, which in the case of lung cancer would be bronchial and alveolar epithelial cells. However, AhR knockdown has also been shown to impair angiogenesis and compromise tumor xenograft growth in mice, by a mechanism involving AhR-dependent VEGF activation in endothelial cells [374]. VEGF is also regulated by the hypoxia-inducible factor-α (HIF-1 α), a PAS family member [375]. Angiogenesis as well as upregulation of the expression of HIF-1 α, ARNT, and VEGF induced by ischemia are enhanced in AhR knockout mice [376]. Indeed, HIF-1 α and AhR crosstalk has been shown to impact both hypoxia-driven gene expression and AhR target genes, presumably via competition for their common dimerization partner, Arnt, as well as by additional mechanisms relevant e.g. for immune cell regulation [377]. Moreover, the role of AhR in VEGF and angiogenesis regulation could be significantly affected by metabolism of PAHs. In fish cell models, both benzo[*k*]fluoranthene and B[*a*]P have been shown to alter expression of hypoxia reporter gene, presumably via their metabolites [378]. Interestingly, in human lung adenocarcinoma A549 cells, B[*a*]P has been found to promote induction of HIF-1 α target genes, including VEGF and carbonic anhydrase IX (CA IX) [379]. Another study indicated that a metabolite of B[*a*]P, B[*a*]P-3,6-dione, can induce HIF-1 α degradation in A549 cells [380]. By contrast BPDE and dihydrodiol epoxide metabolite of chrysene have both been reported to stimulate VEGF induction independently of HIF-1 α [381]. Thus, effects of PAHs on HIF-1a-driven angiogenesis in tumor cells could be regulated not only by their AhR activity but they could be directed also by a pattern of their metabolites being formed in target cells.

## 11. Role of PAHs and AhR in regulation of cancer cell stemness and metastasis

Acquisition of stem cell-like tumor phenotype (cancer stemness) and cancer stem cells are playing an important role in chemoresistance, tumor progression and metastasis. Cancer stem cells have been found to be multidrug-resistant (MDR) based on high expression of the multidrug transporter ATP-binding cassette super-family G member 2 (ABCG2) which is an efflux protein, also called the breast cancer resistance protein (BCRP) [382]. Interestingly, ABCG2 has been identified as a direct transcriptional target of AhR [383]. Consequently, the AhR has been implicated in cancer stemness serving as a sensor and molecular bridge between environmental exposure to PM and PAHs and an increased risk to develop metastases. In the lung, AhR has been shown to induce the expression of ABCG2 and other critical genes involved in cancer stemness [384] which has been found to be associated with an increase of stem population in osteosarcoma cells [385]. Further, the stabilization and activation of AhR has been associated with the expression of deubiquitinase UCHL3 promoting cancer stemness in non-small cell lung carcinoma [386]. The role of AhR in metastasis and cancer stemness seems to be rather complex and may involve various signaling pathways and cell types. Nonetheless, there is increasing evidence that chronic and sustained activation of AhR by environmental toxins (e.g. dioxins and PAHs) promotes carcinogenesis by supporting cancer stemness, chemoresistance and metastasis [364,387].

Atmospheric PM and associated pollutants have been also shown to alter EMT in lung epithelial and bronchial epithelial cell models. EMT plays a central role in various lung diseases, including pulmonary fibrosis and lung cancer. Effects of PM and other particles on EMT have been reviewed extensively in a recent work of Cochard and colleagues [388]. EMT is defined as a process by which cells lose their epithelial phenotype and acquire mesenchymal traits, which include increased ability to migrate and invade. As such, it plays a central role in cancer metastasis. This physiological process occurring during embryogenesis and organ development, which is usually defined by a loss of expression of E-cadherin and acquisition of expression of N-cadherin and vimentin, consists of numerous transition steps, which are only partially recapitulated in cancer cells [389–391]. Nevertheless, already partially executed EMT program may drive cancer metastasis and affects plasticity of tumor cells [389].

Regarding the impact of PM (and PAHs) on EMT in pulmonary cells, numerous studies have been carried out *in vitro* during recent years, and the cellular models used included both bronchial and alveolar epithelial cell models. The treatments included ambient PM_2.5_, DEP, PM derived from biomass burning and a number of standard reference materials (SRM), in both particulate forms and applied as their organic extracts [388]. There is a significant variability in dosing regimens or exposure times, but in general, a wide spectrum of PMs, or their extracts, have been shown to cause EMT in cell models derived from respiratory cells [388]. Studies using PM, DEP and/or individual PAHs as model PAH have indicated that these treatments may cause EMT-like phenotype in alveolar epithelial A549 or in human immortalized bronchial epithelial cells [281,392–395]. Their effects were mostly associated with the loss of E-cadherin expression and increased motility of target cells; nevertheless, the mechanisms underlying these effects remain only partially understood. Interestingly, a two-week exposure to B[*a*]P, but not TCDD, promoted mesenchymal-like phenotype in A549 cells. While TCDD increased the proliferative rate of A549 cells, exposure to B[*a*]P decreased cell proliferation and induced EMT-like phenotype, which was associated with enhanced cell migration, invasion, and altered cell morphology. These changes were mediated by the p21^Cip1^ -dependent delay in cell cycle progression [281]. Thus, activation of the AhR alone was not sufficient to elicit EMT in this cell model.

In human bronchial BEAS-2B cells, a short-term exposure to PM induced matrix metalloproteinase MMP1, extracellular matrix (ECM) remodeling genes, and several other genes related to EMT [392]. PM, cigarette smoke condensate and B[*a*]P have induced EMT in human bronchial epithelial cells (HBEC). However, twelve weeks of chronic exposure to these mixtures or to B[*a*]P were necessary to establish mesenchymal-like phenotype [396]. Deregulation of serpin family B member 2 (SERPINB2) expression is another mechanism that has been suggested to link EMT and PM exposure in human bronchial cells [397]. The upregulation of SERPINB2 via AhR-dependent mechanism [398] induced morphological alterations but it reduced cell migration after short-term exposure to PM2.5; in contrast, in transformed mesenchymal-like HBEC has been strongly SERPINB2 down-regulated. The overexpression of SERPINB2 in PM-exposed bronchial cells might be interpreted as an initial protective mechanism, helping to maintain the epithelial character of the cells [397].

Comparative HPLC-MS/MS analysis of parental HBEC-12KT and B [*a*]P-transformed HBEC-12KT-B1 (the cells with acquired mesenchymal-like phenotype) has revealed significant changes in sphingolipid (SL) and glycosphingolipid (GSL) profiles, favoring those SLs and GSLs which have been reported to act as positive modulators of EMT and other pro-carcinogenic processes [399]. Being both intracellular signaling molecules and important integral components of membrane lipid signaling domains, specific SLs and GSLs have been reported to be involved in cancer development, via playing multiple roles in promoting cancer cell growth and survival, as well as in EMT, cell migration and invasion [400–402]. Interestingly, exosomes isolated from mesenchymal-like HBEC-12KT-B1 cells contained similarly altered SL/GSL profiles indicating a possibility that exosomes derived from transformed mesenchymal-like cells might contribute to cancer progression also in recipient cells [399].

Taken together, multiple mechanisms leading to EMT in airway epithelial cells (both normal and cancer cells) have been reported after exposures to PM, DEP, their extracts or to individual PAHs. The AhR-dependent action of PAHs could also be modified by toxic effects of other PM components [388], leading to generation of oxidative stress, inflammatory responses or disruption of DNA integrity and cell proliferation. Together, these effects may lead to activation of transcription factors regulating EMT response. Overall, the mechanisms underlying induction of mesenchymal-like phenotype in lung epithelium will require further attention, as this mechanism may significantly contribute to dissemination of lung cancer cells and formation of metastases. Another line of evidence supporting this comes from the experiments with cigarette smoke, which contains large quantities of PAHs and AhR ligands, and which has been documented to induce EMT in lung adenocarcinoma A549 cells [403]. The cigarette smoke extract-induced intracellular ROS increased expression of runt-related transcription factor 2 (RUNX-2) and galectin-3, a novel mechanism likely to contribute to EMT induction [403]. The effects of PAHs and their mixtures on EMT are mostly non-genotoxic. They might be relevant for normal cells of respiratory epithelium, during early stages of cell transformation, as well as during cancer progression, where they promote cancer cell dissemination.

## 12. Framework for development of adverse outcome pathways (AOPs) for air pollution induced lung cancer

**Fig. 2.**
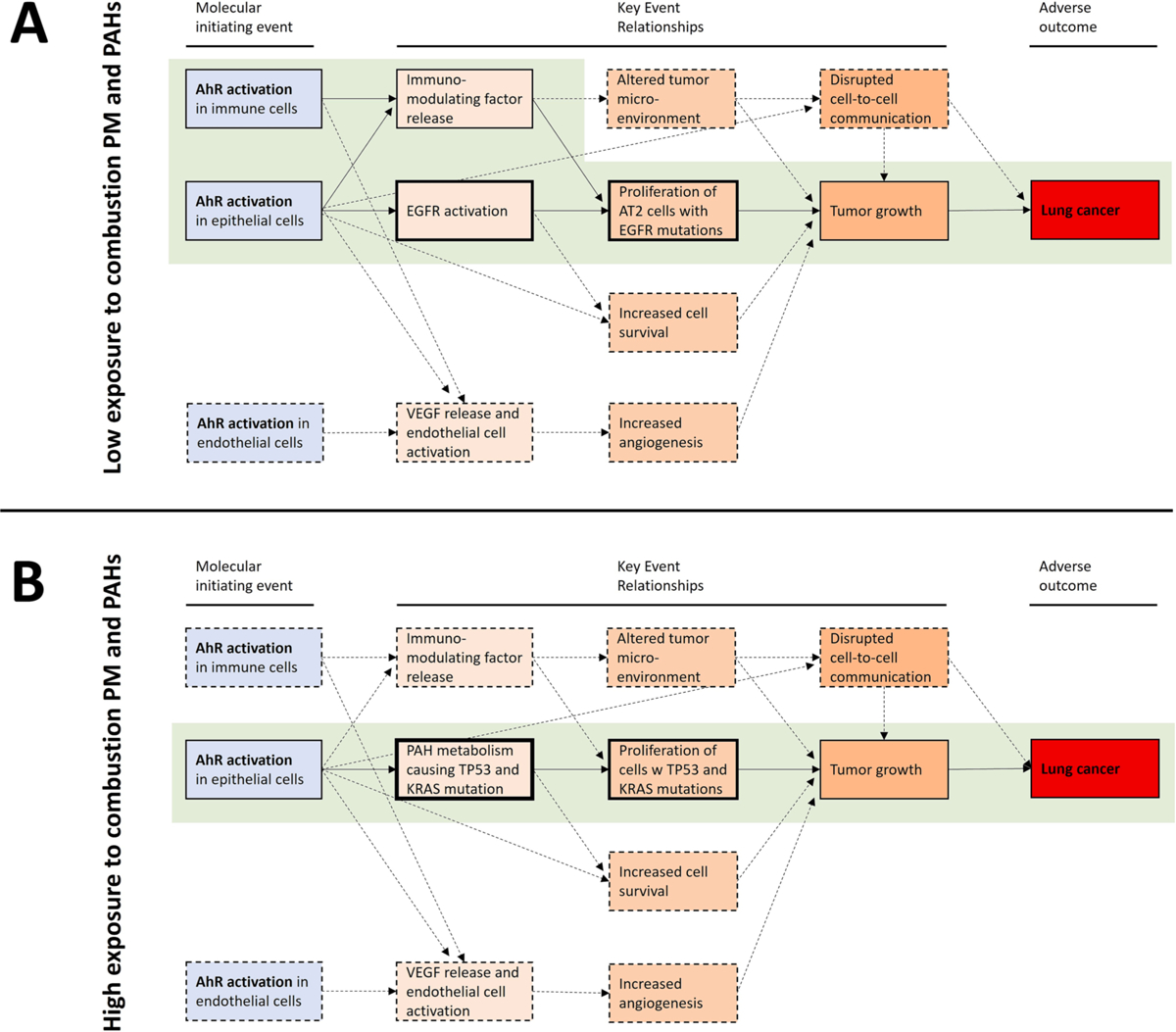
A framework for development of Adverse Outcome Pathways (AOPs) for AhR in lung cancer from air pollution and combustion PM. The figure presents a framework for development of AOPs for the link between AhR activation in different lung cell types and development of lung cancer after exposure of low levels of combustion PM and PAHs from outdoor air (A) and high-level exposures from smoking or occupational settings (B). At low-level exposure (A), AhR activation is primarily suggested to induce lung cancer by tumor promotion, through release of proinflammatory IL-1 family cytokines and nongenomic activation of EGFR. At high-level exposure (B) AhR induced CYP1 expression with subsequent PAH metabolism, formation of genotoxic metabolites and mutations in TP53 and KRAS is believed to be a central, early key events. AhR induced tumor promotion likely also affects cancer development in the high-level exposure scenario, but the role is less clear and suppressive effects of AhR on KRAS-driven cancers have been reported. It should be noted that some of MIE like VEGF release resulting in increased angiogenesis are first of importance in the later stage of cancer development; while key events like release of immunomodulating factors, DNA damage/mutations, increased cell survival, disrupted cell-to-cell-communication are of importance during a much longer period of cancer development than indicated in the figures. *Well documented connections between AhR activation as the molecular initiating event, different key events, and the adverse outcome (lung cancer) are highlighted by solid lines on green background. Dotted lines represent connections that are indicated in the literature but where more uncertainty still exists*.

Recently, an adverse outcome pathway (AOP) was proposed for breast-cancer related cell death, with AhR as the molecular initiating event (MIE), decreased apoptosis and increased motility, inflammation, and endothelial migration as cellular key events (KE) [29]. As discussed in the present review, AhR and PAHs appear to affect many of the same responses in the lungs and a corresponding AOP could likely be developed for lung cancer development from PM_2.5_ and combustion particles. However, the AOP for AhR-induced breast cancer, which was based on an artificial intelligence tool, provides limited molecular insight into the KEs induced by AhR activation in breast cancer cells [29]. By contrast, the recent studies on air pollution induced lung cancer discussed in this review provide a more detailed mapping of the molecular and cellular events contributing to adenocarcinoma development from ambient air PM_2.5_ exposure. Air pollution induced lung cancer in never-smokers appear primarily to be due to promotion of AT2 cells harboring naturally acquired EGFR mutations. The collective evidence suggest that AhR plays a central role by regulating proinflammatory cytokines in various lung cells. Additional evidence for a central role of AhR in EGFR driven lung cancers from combustion particle exposure comes from the well-established link between AhR non-genomic signaling and activation of EGFR, and the observations that AhR nuclear translocation, a marker of AhR activation, is common in lung cancer from never-smokers. Based on this we suggest a framework for the role of AhR in lung cancer development from air pollution and other low concentrations of combustion PM, were AhR activation in macrophages and epithelial cells may represent the MIE leading release of IL-1 family cytokines such as IL-1 β and IL-18, and activation of EGFR which both contributes to induce proliferation of AT2 cells with EGFR driver mutations subsequently leading to tumor growth and lung ADC development (Fig. 2a). Although the role of AhR non-genomic signaling in EGFR activation is well established, it still remains unclear whether and how PM_2.5_ contribute to activation of EGFR with oncogenic mutations. Furthermore, additional AhR-regulated mechanisms clearly contribute to cancer progression through enhancing cell survival/suppression of apoptosis, altered tumor microenvironment, reduction of contact inhibition and increased angiogenesis (Fig. 2a). At higher combustion PM exposure doses, AhR-induced PAH metabolism and mutations in particular in TP53 and KRAS, become more important (Fig. 2b). Additional effects of AhR activation on inflammation, tumor microenvironment, cell-to-cell communication, cell proliferation and survival, are likely to occur also in these cases. However, it should be noted that proliferation and colony formation of lung cancer cells with KRAS mutations may be less affected by PM2.5 exposure than lung cancer cells harboring tEGFR mutations, and AhR has also been reported to suppress KRAS-driven NSCLC. It should also be considered that other combustion-derived mutagens not discussed in this review (e.g. aldehydes, nitrosamine, metals, and ultrafine-/nanoparticles as such) may contribute significantly to lung cancer development at high combustion PM exposure.

Conclusive evidence for the role of AhR and PAHs in many of these processes is still lacking. However, this suggested framework for AhR signaling in lung cancer may provide a guidance for future studies and development of AOPs for AhR in lung cancer from exposure to ambient air PM_2.5_ and combustion PM. For instance, there is a need to explore how AhR knockdown or pharmacological inhibition would affect PM_2.5_ induced tumor promotion in lung AT2 cells with EGFR driver mutations and to compare the impact of PM_2.5_ with high or low PAH content on these responses. It is, however, important to consider that the role of AhR in lung cancer development is highly complex and, as is often the case in AhR research, that contrasting findings have been reported. The key to understanding the apparent multifaceted role of AhR in tumor development may lie in the diversity of responses regulated by unliganded constitutively active AhR, and upon distinct modes of AhR activation being elicited by its different ligands. Activation of AhR by PAHs and other ligands does not merely function as an on–off switch for transcription of target genes. AhR rather appears to bind and regulate a large number of gene clusters in unstimulated cells, and ligand-dependent activation causes considerable qualitative shifts in the genes regulated by the receptor [304]. A similar ligand promiscuity has also been described for the non-genomic effects of AhR [214,233]. These qualitative shifts in signaling and responses could likely explain some of the apparent contradictory results reported from studies on AhR in lung cancer based on knockout or overexpression models versus those based on exposure to different AhR ligands. Moreover, AhR could play specific roles in different types of lung cancers, where some express high AhR levels and others do not, and some are induced by AhR, while others are suppressed by AhR activity. Clarifying the underlying mechanisms for this “Janus-faced” role of AhR in lung cancer will be important. Another central question relates to the dose–response (or concentration-effect) relationship between PM or PAH exposure and different responses regulated by the AhR. The wide range of cellular processes regulated by AhR are presumably activated at somewhat different dose levels. Identifying the most sensitive biological responses induced by AhR may provide important information on the main mechanisms driving lung cancer development at relatively low PM-exposure levels encountered in outdoor air. After all, activation of AhR appears to be among the most sensitive endpoints reported from *in vitro* exposure of lung cell models to PM or DEP [111,112].

## 13. Conclusion

After more than half a century of research originating from studies on PAH metabolism, our understanding of the role of AhR in cancer development has expanded dramatically. For lung cancer, as for many other cancer types, AhR has been implicated at all stages of tumor development including initiation, promotion, progression, invasion, and metastasis.

We propose that lung cancer from smoking (and occupational and domestic exposure to high combustion PM levels) and lung cancer from air pollution (and secondhand smoke) in never-smokers represent the two ends of a dose–response continuum (Fig. 2a and b). In the case of lung adenocarcinomas (ADC) development in never-smokers from PM_2.5_ exposure from air pollution, tumor promotion appears to be a key mechanism acting on lung cells with EGFR driver-mutations acquired naturally through ageing. PAHs from combustion PM are likely candidate components contributing to these responses, through AhR-mediated activation of IL-1 family cytokines such as IL-1β and IL-18 induced through genomic pathways, and possibly also through non-genomic activation of EGFR. Moreover, AhR signaling upregulates immune-regulatory factors and can generate a pro-tumorigenic microenvironment enabling tumor promotion as discussed in this review. For lung squamous cell carcinoma (SCC) development in the central airways induced by higher exposure levels of combustion PM from smoking, occupational exposure, or indoor coal combustion, the initiation step appears to be a key mechanism driven by mutagenic PAH-metabolites through the classical AhR:Arnt-CYP pathway, acting in combination with other combustion-derived mutagens. The tumor promoting effects of AhR may also be involved in SCC, but they might be less prominent here. Accordingly, AhR has been reported to suppress some lung cancers, including those with KRAS-driver mutations characteristic of PAH-induced genotoxicity and smoking.

Clarifying the role of AhR in lung cancer development associated with air pollution and combustion PM may provide tools for detecting vulnerable populations and give a deeper understanding of essential risk factors. Hopefully this will lead to more efficient measures to reduce exposure to the most harmful air pollutants which can help to intervene and mitigate the development of cancer, especially for people at higher risk through environmental exposure to air pollution.

## Abbreviations:

ATF4: Activating transcription factor 4
ADC: adenocarcinomas
AJs: adherens junctions
AT2: alveolar type 2
AOP: adverse outcome pathway
ALK: anaplastic lymphoma kinase
AR: androgen receptor
APAF1/Apaf1: apoptotic peptidase activating factor 1
Arg1: arginase 1
AhR: aryl hydrocarbon receptor activities
REPs: AhR-activating relative potencies
ARA9 or XAP2: AhR-interacting protein
Arnt: AhR nuclear translocator
ABCG2: ATP-binding cassette super-family G member 2
β-ARs: beta-adrenergic receptors
B[*a*]P: benzo[*a*]pyrene
BPDE: B[*a*]P-7,8-dihydrodiol-9,10-epoxide
BPDE-N2-Dg: B[a]P 7,8-diol-9,10-epoxide-N2-deoxyguanosine
BRAF: B-Raf proto-oncogene
BCRP: breast cancer resistance protein
BALFs: bronchoalveolar lavage fluids
CHP2: calcineurin homologous protein isoform 2
CA IX: carbonic anhydrase IX
Cav1: caveolin-1
KE: cellular key events
CHK2: checkpoint kinase-2
CXCL8: chemokine CXC-motif ligand 8
COPD: chronic obstructive pulmonary disease
CAR: constitutive androstane receptor
COX-2: cyclooxygenase 2
CYP: cytochrome P450
DEP: diesel exhaust particles
DREs: dioxin response elements
DTT: dithiothreitol
EC: elemental carbon
(EoL-1) cells: eosinophilic
EGFR: epidermal growth factor receptor
EGFR-TKI: EGFR tyrosin kinase inhibitor
EMT: epithelial-mesenchymal transition
EGFR TKI: EGFR tyrosine kinase inhibitor
ER: estrogen receptors
ERR1/2: extracellular regulated kinase
ECM: extracellular matrix
EVs: extracellular vesicles
EOM: extractable organic material
FGF-2: fibroblast growth factor 2
GJs: gap junctions
GJIC: gap junctional intercellular communication
GBD: Global Burden of Disease
GR: glucocorticoid receptor
GST: glutathione S-transferase
GSL: glycosphingolipid
Hsp90: heat shock protein 90 dimer
MET: hepatocyte growth factor receptor
HBEC3-KT: human bronchial epithelial cells
BEAS-2B: human bronchial epithelial cell
HER2: human epidermal growth factor receptor 2
HUVECs: human umbilical vein endothelial cells
HIF-1a: hypoxia-inducible factor-a
IDO: indoleamine 2,3-dioxygenase
IL: interleukin
LDH: intracellular calcium concentrations [Ca^2+^]_*i*_ lactate dehydrogenase
LKB-1: liver kinase B1
L-Kyn: L-kynurenine
mTOR: mammalian target of rapamycin
MV: microvesicles
MEK-1: mitogen activated protein/extracellular regulated kinase kinase
MAPK: mitogen activated kinase
mdm2: mouse double minute 2
MDR: multidrug-resistant
MDSCs: myeloid derived suppressor cells
NQO1: NADPH:quinone oxidoreductase
nitro-PAHs: nitrated PAHs
1-NP: 1-nitropyrene
ncRNAs: noncoding RNAs
miRNA: microRNA
NSCLC: non-small cell lung cancer
NF-kB: nuclear factor-kB
OR: odds ratio
OC: organic carbon
8-oxoG: 8-oxo-7,8-dihydro-guanine
oxy-PAHs: oxygenated PAHs
PM: particulate matter
EOM: PM-extractable organic material
PTEN: phosphatase with tensin homology
PAHs: polycyclic aromatic hydrocarbons
PCBs: polychlorinated biphenyls
Poly I:C: polyinosinic:polycytidylic acid
PR: progesterone receptor
PD-1: programmed cell death protein 1
PD-L1: PD-1 ligand
WB-F344: rat liver epithelial cells
ROS: reactive oxygen species
RET: rearranged during transfection
RelBAHRE: RelB/AhR response element
PM-EOM: residual particles after the extractions
RUNX-2: runt-related transcription factor 2
SERPINB2: serpin family B member 2
SCLC: small-cell lung cancer
NHE: sodium hydrogen exchanger Na^+^/H^+^ exchanger
SL: sphingolipid
p65: RelA and RelB, subunits of NF-kB
SRM: standard reference material
cancer stemness: stem cell-like tumor phenotype
SCC: squamous cell carcinoma
SHS: secondhand smoke
TCDD: 2,3,7,8-tetrachlorodibenzo-p-dioxin
TEFs: toxic equivalency factors
TJs: tight junctions
TMPRSS2: transmembrane serine protease 2
TRPC: transient receptor potential canonical
TAMs: channels,tumor associated macrophages
TME: tumor microenvironment
TNF: tumor necrosis factor
TP53: -α,tumor protein 53
TP73/p73: tumor protein 73
TRCs: tumor-repopulating T-cells
UGT: tyrosine protein kinase c-Src,UDP-glucuronosyltransferase
VEGF: vascular endothelial growth factor
KRAS: v-Ki-ras2 Kirsten rat sarcoma viral oncogene homolog
WSP: wood smoke particles
XRE/DRE: xenobiotic or dioxin response elements
